# Quantifying chromosomal instability from intratumoral karyotype diversity using agent-based modeling and Bayesian inference

**DOI:** 10.7554/eLife.69799

**Published:** 2022-04-05

**Authors:** Andrew R Lynch, Nicholas L Arp, Amber S Zhou, Beth A Weaver, Mark E Burkard

**Affiliations:** 1 Carbone Cancer Center, University of Wisconsin-Madison Madison United States; 2 McArdle Laboratory for Cancer Research, University of Wisconsin-Madison Madison United States; 3 Department of Cell and Regenerative Biology, University of Wisconsin Madison United States; 4 Division of Hematology Medical Oncology and Palliative Care, Department of Medicine University of Wisconsin Madison United States; University of Edinburgh United Kingdom; Utrecht University Netherlands

**Keywords:** aneuploidy, single-cell sequencing, agent-based modeling, mitosis, approximate Bayesian computation, Human

## Abstract

Chromosomal instability (CIN)—persistent chromosome gain or loss through abnormal mitotic segregation—is a hallmark of cancer that drives aneuploidy. Intrinsic chromosome mis-segregation rate, a measure of CIN, can inform prognosis and is a promising biomarker for response to anti-microtubule agents. However, existing methodologies to measure this rate are labor intensive, indirect, and confounded by selection against aneuploid cells, which reduces observable diversity. We developed a framework to measure CIN, accounting for karyotype selection, using simulations with various levels of CIN and models of selection. To identify the model parameters that best fit karyotype data from single-cell sequencing, we used approximate Bayesian computation to infer mis-segregation rates and karyotype selection. Experimental validation confirmed the extensive chromosome mis-segregation rates caused by the chemotherapy paclitaxel (18.5 ± 0.5/division). Extending this approach to clinical samples revealed that inferred rates fell within direct observations of cancer cell lines. This work provides the necessary framework to quantify CIN in human tumors and develop it as a predictive biomarker.

## eLife digest

DNA contains all the information that cells need to function. The DNA inside cells is housed in structures called chromosomes, and most healthy human cells contain 23 pairs. When a cell divides, all chromosomes are copied so that each new cell gets a complete set. However, sometimes the process of separating chromosomes is faulty, and new cells may get incorrect numbers of chromosomes during cell division. Cancer cells frequently exhibit this behavior, which is called chromosomal instability’, or CIN.

Chromosomal instability affects many cancer cells with varying severity. In cancers with high chromosomal instability, the number of chromosomes may change almost every time the cells divide. These cancers are often the most aggressive and difficult to treat.

Scientists can estimate chromosomal instability by counting differences in the number of chromosomes across many cells. However, many cells that are missing chromosomes die, resulting in inaccurate measures of chromosomal instability. To find a solution to this problem, Lynch et al. counted chromosomes in human cells with different levels of chromosomal instability and created a computer model to work out the relationship between chromosomal instability and chromosome number.

The model could account for both living and dead cells, which gave more accurate results. Lynch et al. then confirmed the accuracy of their approach by using it on a group of cells treated with a chemotherapy drug that causes a known level of chromosomal instability. They also used existing data from breast and bowel cancer, which revealed that levels of chromosomal instability varied between one mistake per three to twenty cell divisions.

Lower levels of chromosomal instability can be linked to a better prognosis for cancer patients, but it currently cannot be measured reliably. These results may help to reveal the causes of chromosomal instability and the role it has in cancer. If this method is successfully applied to patient samples, it could also improve our ability to predict how each cancer will progress and may lead to better treatments.

## Introduction

Chromosomal instability (CIN) is characterized by persistent whole-chromosome gain and loss through mis-segregation during cell division. Genome instability is a hallmark of cancer (Hanahan and Weinberg, 2011) and one type, CIN, is the principal driver of aneuploidy, a feature of ~80% of solid tumors (Hancock et al., 2004; Knouse et al., 2017; Weaver and Cleveland, 2006). CIN potentiates tumorigenesis (Foijer et al., 2017; Levine et al., 2017; Silk et al., 2013) and associates with therapeutic resistance (Ippolito et al., 2020; Lee et al., 2011; Lukow et al., 2020; Pavelka et al., 2010), metastasis (Bakhoum et al., 2018) and poor survival outcomes (Bakhoum et al., 2011; Denu et al., 2016; Jamal-Hanjani et al., 2017). Thus, CIN is an important characteristic of cancer biology. Despite its importance, CIN has not emerged as a clinical biomarker, in part because it is challenging to quantify.

Although CIN has classically been characterized as binary—tumors either have it or not—recent evidence highlights the importance of the rate of chromosome mis-segregation and the specific aneuploidies it produces. For example, clinical outcomes partially depend on aneuploidy of specific chromosomes (Davoli et al., 2013; Sheltzer et al., 2017; Vasudevan et al., 2020). Further, higher levels of CIN suppress tumor growth when they surpass a critical threshold, thought to be due to lethal loss of essential genes and irregular expression due to imbalanced gene dosage (Funk et al., 2021; Silk et al., 2013; Weaver and Cleveland, 2008; Zasadil et al., 2014). Moreover, baseline CIN may predict chemotherapeutic response to paclitaxel (Janssen et al., 2009; Swanton et al., 2009) and is proposed to both promote detection by or evasion from the immune system (Davoli et al., 2017; Santaguida et al., 2017). No single or standardized analytically valid measure of CIN has emerged and this gap has precluded its clinical validation as a prognostic or predictive biomarker.

Prior measures of CIN use various means to compare levels in tumors or populations, but do not establish a standardized quantitative rate. These prior measures include histologic analysis of mitotic defects (Bakhoum et al., 2011; Jin et al., 2020), fluorescence in situ hybridization (FISH) with probes to detect individual chromosomes (Thompson and Compton, 2008), and gene-expression methodologies like CIN scores (Carter et al., 2006). While these methods are readily accessible, they have significant drawbacks for clinical application. FISH and mitotic visualization approaches are laborious. Direct visualization of mitotic defects to measure CIN is only possible in the most proliferative tumors where enough cells are captured in short-lived mitosis. FISH typically quantifies only a subset of chromosomes, which will be misleading if there is bias toward specific chromosome gains/losses (Dumont et al., 2020). While gene expression scores are proposed as indirect measures of CIN, they are not specific to CIN and correlate highly with proliferation and structural aneuploidy (Carter et al., 2006; Sheltzer, 2013).

Single-cell sequencing promises major advances in quantitative measures of CIN by displaying cell-cell variation for each chromosome across hundreds of cells (Navin et al., 2011; Wang et al., 2014). However, selection poses another complication. To date, single-cell analyses have identified surprisingly low cell-cell karyotype variation, even when mitotic errors are directly observed by microscopy (Bolhaqueiro et al., 2019; Gao et al., 2016; Kim et al., 2018; Nelson et al., 2020; Wang et al., 2014). These observations highlight the confounding role of selection against aneuploid karyotypes in measuring CIN in human tumors. Indeed, selection reduces karyotype variance in cancer cell populations that directly exhibit mitotic errors (Gerstung et al., 2020; Ippolito et al., 2020; Lukow et al., 2020). Here, we seek to overcome gap by modeling chromosomal instability and explicitly considering the evolutionary selection of aneuploid cells, to derive a quantitative measure.

We describe a quantitative framework to measure CIN by sampling population structure and cell-cell karyotypic variance in human tumors, accounting for selection on aneuploid karyotypes. We built our framework on the use of phylogenetic topology measures to quantify underlying evolutionary processes (Mooers and Heard, 1997); in this case to quantify CIN from both the diversity and the aneuploid phylogeny within a tumor. Using an agent-based model of CIN, we determine how distinct types and degrees of selective pressure shape the karyotype distribution and population structure of tumor cells at different rates of chromosome mis-segregation. We then use this in silico model as a foundation for parameter inference to provide a quantitative estimate of CIN as the numerical rate of chromosome mis-segregation per cell division. We apply this model to quantify CIN caused by the chemotherapeutic paclitaxel in culture. Next, using existing single-cell whole-genome sequencing data (scDNAseq), we measure CIN in cancer biopsy and organoid samples. As a whole, this work provides a framework to quantify CIN in human tumors, a first step toward developing CIN as a prognostic and predictive biomarker.

## Results

### A framework for modeling CIN and karyotype selection

To assess intratumoral CIN via cell-cell karyotype heterogeneity, we considered how selection on aneuploid karyotypes impacts observed chromosomal heterogeneity within a tumor. By modeling fitness of aneuploid cells, we observe chromosomal variation in a population of surviving cells. The selective pressure of diverse and specific aneuploidies on human cells has not been, to our knowledge, directly measured. Therefore, we employ previously developed models of selection.

In models of CIN, fit karyotypes are selected while unfit aneuploid karyotypes are eliminated over time (Ippolito et al., 2020; Ravichandran et al., 2018; Sheltzer et al., 2017; Vasudevan et al., 2020). We use two previously proposed models of aneuploidy-associated cellular fitness, as well as hybrid and neutral selection models. The Gene Abundance model is based on the relatively low incidence of aneuploidy in normal tissues and assumes cellular fitness declines as the cell’s karyotype diverges from a balanced euploid karyotype (Sheltzer and Amon, 2011; Zhu et al., 2012). When an individual chromosome diverges from euploid balance (2 N, 3 N, 4 N, for example), its contribution to cellular fitness is weighted by its abundance of genes (Figure 1—figure supplement 1A, left). Alternatively, the Driver Density model assumes that each chromosome’s contribution to cellular fitness is weighted by its ratio of Tumor suppressor genes, Oncogenes, and Essential genes (TOEs)(Davoli et al., 2013; Laughney et al., 2015). For example, Driver Density selection will favor loss of chromosomes with many tumor suppressors and favor gain of chromosomes replete with oncogenes and essential genes (Figure 1—figure supplement 1A, right). The hybrid averaged model accounts for both karyotypic balance and TOE densities (Figure 1—figure supplement 1A, middle). Using these fitness models, we assigned chromosome scores to reflect each chromosome’s value to cellular fitness (Figure 1—figure supplement 1B, Table 1), the sum of which represent the total fitness value for the cell, relative to a value of 1 for a euploid cell. Further, we scaled the impact of cell fitness with a scaling factor, S, ranging from 0 (no selection) to 100 (high selection). While these models are approximations, they are nevertheless useful to estimate how mis-segregation and selective pressure cooperate to mold karyotypes in the cell population.

**Table 1.** Base chromosome-specific fitness scores for individual models.

	Selection model		
CHR ARM	Gene Abundance	Driver Density	Hybrid
1p	0.04780162	–0.0024018	0.02269992
1q	0.04340321	0.03244362	0.03792341
2p	0.02733655	0.02935717	0.02834686
2q	0.04244054	0.03943267	0.0409366
3p	0.02310412	0.03289695	0.02800053
3q	0.0299756	0.05416736	0.04207148
4p	0.01238195	0.01784909	0.01511552
4q	0.03181796	0.02901324	0.0304156
5p	0.01178443	0.04281166	0.02729805
5q	0.03787615	0.01949934	0.02868775
6p	0.02557719	0.02398619	0.02478169
6q	0.02554399	0.00011625	0.01283012
7p	0.0179588	0.09889284	0.05842582
7q	0.03231589	0.06933314	0.05082451
8p	0.01591728	0.02769564	0.02180646
8q	0.0254942	0.05861427	0.04205423
9p	0.01301266	–0.0012941	0.00585929
9q	0.02572657	0.04702681	0.03637669
10 p	0.0112201	–0.0364218	–0.0126008
10q	0.02750253	0.01142688	0.01946471
11 p	0.01961858	0.03818621	0.0289024
11q	0.03629936	0.01898784	0.0276436
12 p	0.0142575	0.0551551	0.0347063
12q	0.03659812	0.06273786	0.04966799
13 p	0	0	0
13q	0.02333649	–0.0101539	0.00659128
14 p	1.66E-05	0	8.30E-06
14q	0.03792594	0.02557439	0.03175016
15 p	0	0	0
15q	0.03701306	0.0206566	0.02883483
16 p	0.02383442	0.04334736	0.03359089
16q	0.01900446	–0.0071444	0.00593005
17 p	0.01548573	–0.0085975	0.00344414
17q	0.03553586	0.04363474	0.0395853
18 p	0.00627396	0.00533697	0.00580547
18q	0.01434049	–0.0263632	–0.0060113
19 p	0.02159372	0.05371416	0.03765394
19q	0.02813325	0.00550338	0.01681831
20 p	0.0089628	0.04351025	0.02623653
20q	0.01526996	0.04993593	0.03260295
21 p	0.00232369	0	0.00116185
21q	0.01233215	–0.0033092	0.00451147
22 p	0.00013278	0	6.64E-05
22q	0.02297134	–0.0051581	0.0089066
Xp	0.01555213	0	0.00777606
Xp	0.02499627	0	0.01249813

**Figure 1.**
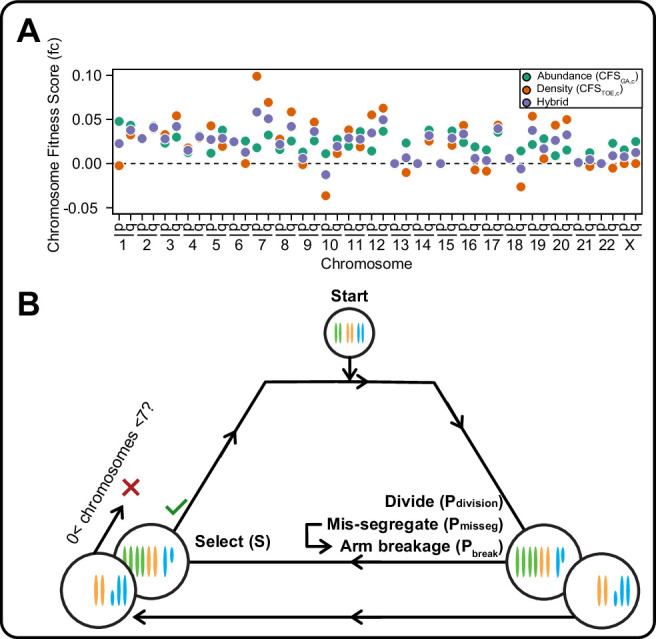
A framework for modeling CIN and karyotype selection. (**A**) Chromosome arm scores for each model of karyotype selection. Gene Abundance scores are derived from the number of genes per chromosome arm normalized to the number of all genes. Chromosome arms 13 p and 15 p did not have an abundance score and were set to 0. Driver Density scores come from the pan-cancer chromosome arm scores derived in Davoli et al., 2013, and normalized to the sum of chromosome arm scores for chromosomes 1-22,X. Chromosome arms 13 p, 14 p, 15 p, 21 p, 22 p, and chromosome X did not have driver scores and were set to 0. Hybrid model scores are set to the average of the Driver and Abundance models. The neutral model (not displayed) is performed with all cell’s fitness constitutively equal to 1 regardless of karyotype. (**B**) Framework for the simulation of and selection on cellular populations with CIN. Cells divide (Pdivision starts at 0.5 in the exponential pseudo-Moran model and is constitutively equal to 1 for the constant Wright-Fisher model) and probabilistically mis-segregate chromosomes (Pmisseg ∈ [0, 0.001… 0.05]). After, cells experience selection under one of the selection models, altering cellular fitness and the probability (Pdivision) a cell will divide again (green check). Additionally, cells wherein the copy number of any chromosome falls to zero or surpasses 6 are removed (red x). After this, the cycle repeats. See Materials and methods for further details.

We employed these selection models in an agent-based model of exponential population growth wherein each cell has its own karyotype (Figure 1 and Figure 1—figure supplement 1). Briefly, simulations started with 100 euploid cells and were run in discrete time steps with variable rates of selective pressure, S, and rates of chromosome mis-segregation (P_misseg_, see definitions in Table 2). The rate—or probability—of mis-segregation events, P_misseg_, is the measure of CIN. During each time step, cells have a P_division_ ( = 0.5 for euploid) chance of dividing. Each dividing cell has a P_misseg_ chance of improper segregation of each chromosome. Segmental chromosome breaks occur with a probability P_break_, set at 0 or 0.5. After division, fitness (F) of each daughter is assessed. Cells are removed from the population if any given chromosome has copy number 0 or >6. The P_division_ value of the remaining viable cells is adjusted by the cell’s fitness under selection (F^S^). Due to computational limitations, pseudo-Moran or Wright-Fisher models are employed to limit the modeled cell population (Figure 1—figure supplement 1C, D). These limits did not significantly affect the measures extracted from these populations (Figure 1—figure supplement 2). Thus, these models simulate an evolving population of aneuploid cells under given rates of CIN, P_misseg_, and models and strength of selection.

**Table 2.** Parameters varied during agent-based modeling.

Parameter	Description
Pmisseg	Probability of mis-segregation per chromosome per division
Pbreak	Probability of chromosome breakage after mis-segregation
Pdivision	Probability of cellular division per time step
S	Magnitude of selective pressure on aneuploid karyotypes

**Figure 1—figure supplement 1.**
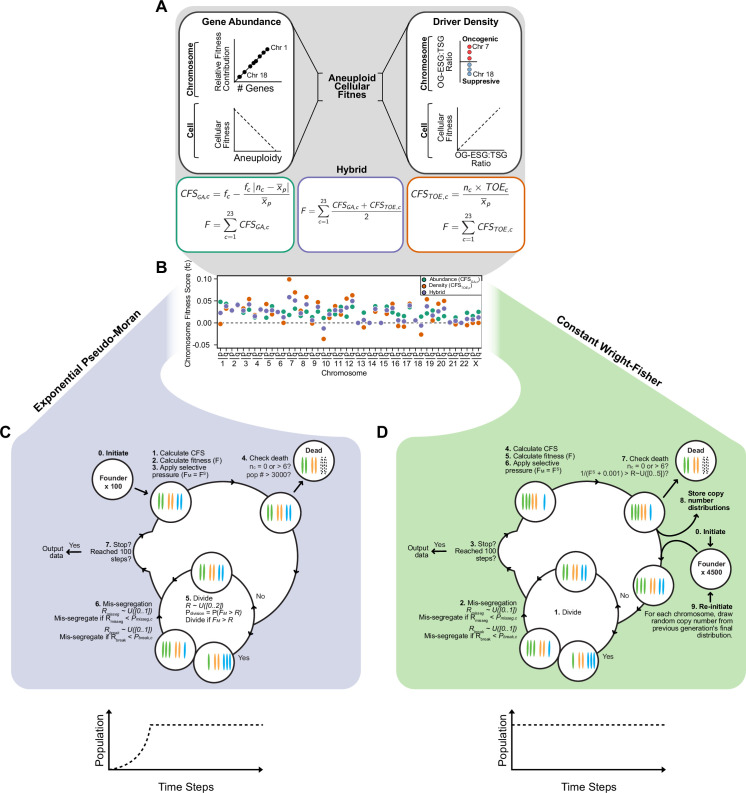
Expanded model of chromosome mis-segregation and karyotypic selection. Models of selection on aneuploid karyotypes. **Left**. In the Gene Abundance model, chromosomes that encode a larger number of genes contribute more to cellular fitness (F). Thus, large chromosomes have a higher fitness score (f_c_). Deviation from the average ploidy of the population results in a reduced Contextual Fitness Score (CFS) for each chromosome, the sum of which represents the fitness of the cell. **Right**. In the Driver Density Model, the fitness contribution of a chromosome depends on the ratio of oncogenes and essential genes to tumor suppressors (OG-ESG:TSG). Gaining chromosomes with a higher OG-ESG:TSG ratio provides a fitness advantage while gaining more suppressive chromosomes invokes a fitness cost. These scores are still normalized to the ploidy of the average ploidy of the population to ensure that higher ploidy populations are not arbitrarily more fit. **Middle**. The Hybrid model takes the average of the fitness scores calculated in the other models. The neutral selection model (not shown) treats all karyotypes as equally fit. Base chromosome arm fitness scores for each model. Only the Hybrid and Driver Density model have negatively scored chromosomes, meaning their loss provides a fitness benefit. The neutral selection model does not require chromosome arm fitness scores. Simulating CIN in exponentially growing populations with pseudo-Moran limits. (**0**) Populations are founded by 100 founder cells and the simulation is initiated. (**1**) CFS values are calculated for each chromosome in a cell according to the chosen model. (**2**) Cellular fitness is calculated based on CFS values. (**3**) Selective pressure (S) is applied on cellular fitness values (F). (**4**) Cells are checked to see if any death conditions are met and if the population limit is met. (**5**) Cells probabilistically enter mitosis if their fitness value exceeds a random float (R) between 0 and 2. Thus P_division_ = P(F_M_ >R). If a cell does not divide, it skips the next step. (**6**) If a cell enters mitosis, each chromosome has an opportunity to mis-segregate probabilistically. For each chromosome, a mis-segregation occurs if a random float (R), from 0 to 1, falls below P_misseg_. After a chromosome mis-segregation is determined, the chromosome arms may be individually segregated (i.e. reciprocal CNA) if a random float (R), from 0 to 1, falls below P_break_. The cycle repeats and new CFS values are calculated, unless (**7**) stop conditions are met. When populations reach or exceed 3500 cells, a random half of the population is eliminated and the remaining cells continue the cycle. Simulating CIN in constant-size populations with Wright-Fisher dynamics. (**0**) Populations are initiated by 4500 euploid cells which (**1**) divide every step. (**2**) Chromosomes are mis-segregated as in the exponential pseudo-Moran model described above. (**3**) If stop conditions are met, the simulation ends and data are exported. If the cycle continues, (**4**) CFS values are calculated and used to (**5**) determine cellular fitness, after which, (**6**) selective pressure is applied. (**7**) Cells die if they lose both copies of a chromosome or exceed the upper limit of six. Additionally, to approximate Wright-Fisher dynamics, cells die if 1/(F^S^ +0.001) exceeds a random float from 0 to 5. Thus, the baseline rate of cell death is ~0.2. (**8**) Each chromosome copy number is stored and the population is re-initiated with 4500 new cells. The copy numbers for each of new cell’s chromosomes are randomly and independently drawn from the copy number distributions of the previous generation. The cycle then repeats until the simulation ends (step 3).

**Figure 1—figure supplement 2.**
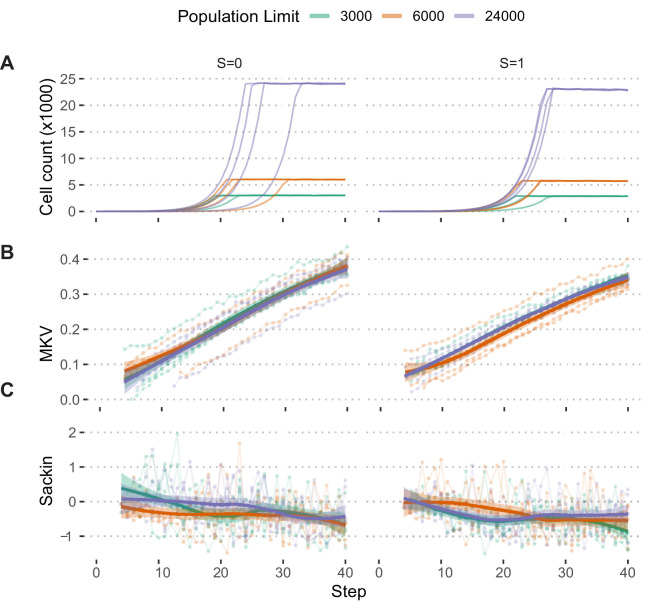
Population growth limits do not bias population measures. (**A**) Growth curves of populations simulated under the Hybrid selection model and exponential pseudo-Moran growth model with S ∈[0,1] and Pmisseg _misseg_ = 0.022 and limited to 3000, 6000, and 24,000 cells (n = 4 simulations each). (**B**) MKV (normalized to mean ploidy of the population) values steadily increase over time. (**C**) Loess regression curves show no significant deviations based on the population threshold, regardless of selection. Tree-tip-normalized Sackin index values for each population over time. No significant deviations based on the population threshold, regardless of selection.

### Evolutionary dynamics is imparted by CIN

**Figure 2.**
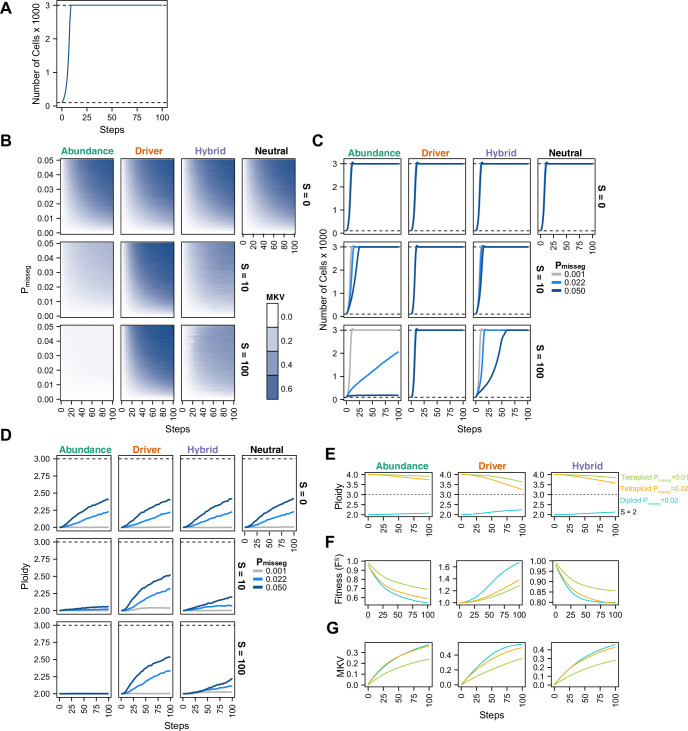
Evolutionary dynamics imparted by CIN. (**A**) Population growth curve in the absence of selective pressure (P_misseg_ = 0.001, S = 0, n = 3 simulations). The steady state population in null selection conditions is 3000 cells. (**B**) Heatmaps depicting dynamics of karyotype diversity as a function of time (steps), mis-segregation rate (P_misseg_), and selection (S) under each model of selection. Columns represent the same model; rows represent the same selection level. Mean karyotype diversity (MKV) is measured as the variance of each chromosome averaged across all chromosomes 1–22, and chromosome X. Low and high MKV are shown in white and blue respectively (n = 3 simulations for every combination of parameters). (**C**) Population growth under each model, varying P_misseg_ and S. P_misseg_∈ [0.001, 0.022, 0.050] translate to about 0.046, 1, and 2.3 mis-segregations per division respectively for diploid cells. (**D**) Dynamics of the average ploidy (total # chromosome arms / 46) of a population while varying P_misseg_ and S. (**E**) Dynamics of ploidy under each model for diploid and tetraploid founding populations. P_misseg_∈ [0.01, 0.02] translate to about 0.46 and 0.92 mis-segregations for diploid cells and 0.92 and 1.84 mis-segregations for tetraploid cells. (**F**) Fitness (F^S^) over time for diploid and tetraploid founding populations evolved under each model. (**G**) Karyotype diversity dynamics for diploid and tetraploid founding populations. MKV is normalized to the mean ploidy of the population at each time step. Plotted lines in C-G are local regressions of n = 3 simulations.

To understand the interplay between CIN and selection, we simulated 100 steps of cell growth with CIN under each selection model. We varied the rate of CIN (P_misseg,c_ ∈[0, 0.001… 0.05] per chromosome; or 0–2.3 chromosome mis-segregations per division) and selective pressure ranging from none to heavy selection (S ∈[0, 2… 100]). As expected, the simulated cell number increases rapidly to the pseudo-Moran cap of 3000, where it remains (Figure 2A). As displayed in Figure 2B, diversity of the cell population, expressed as mean karyotypic variance increases over time, but also depends on mis-segregation rate, and selection levels (Figure 2B). As expected, high mis-segregation rates (P_misseg_, Y axis) and low selection (S = 0; top row) enhance the variance of the population. Further, without selection (S = 0; top row) all models returned comparable profiles over time, resembling neutral selection. However, when selective pressure is applied (S > 0), the distinct profiles appear. The abundance model (first column) negatively selects against all aneuploid karyotypes and yields low heterogeneity that increases modestly with mis-segregation rate. With the Driver model (second column), there is a sharp increase in heterogeneity even at low mis-segregation rates, as this model favors specific aneuploid states that maximizes oncogenes and minimizes tumor suppressors. The Hybrid model falls between the other two. Results were not specific to the pseudo-Moran process of capping at 3000 cells—dynamics were similar in the constant-population Wright-Fisher model (Figure 2—figure supplement 1A, B). These data illustrate how CIN and selection operate together to shape the karyotype diversity in the cell population.

**Figure 2—figure supplement 1.**
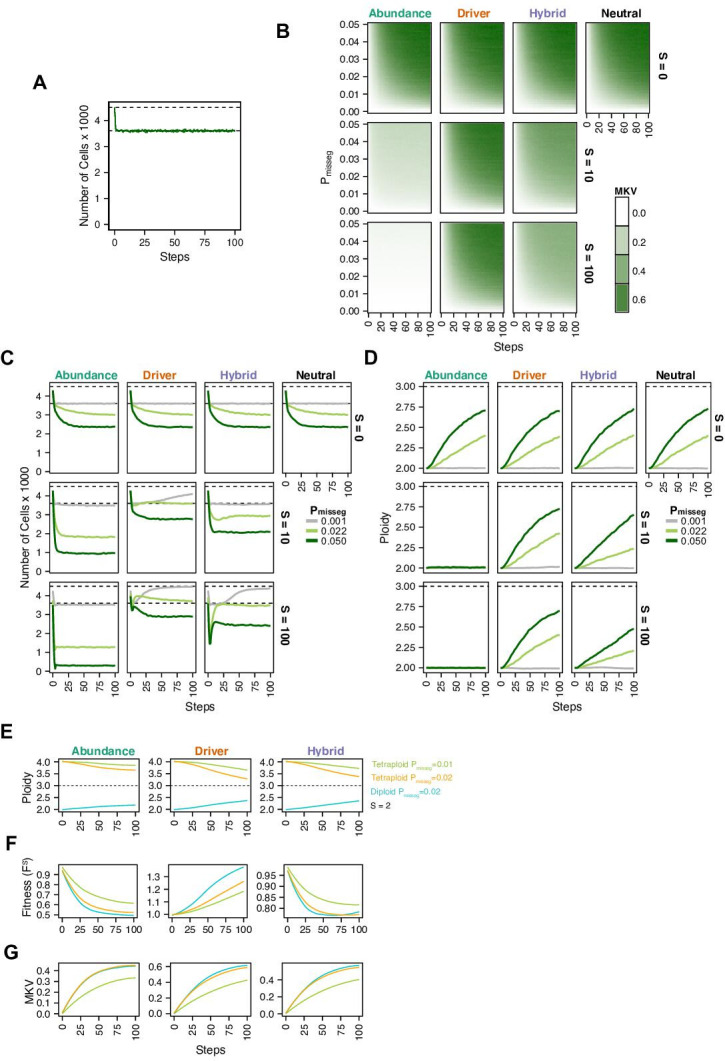
Chromosomal instability and karyotype selection in constant-size populations approximating Wright-Fisher dynamics. (**A**) Population size over time in the absence of selective pressure (P_misseg_ = 0.001, S = 0, n = 3 simulations). The steady state population in null selection conditions is ~3600 cells as data is exported before populations are re-initiated. Dashed line represents the population at (re-)initiation (4500 cells). (**B**) Heatmaps depicting dynamics of karyotype diversity as a function of time (steps), mis-segregation rate (P_misseg_), and selection (S) under each model of selection. Columns represent the same model; rows represent the same selection level. Mean karyotype diversity (MKV) is measured as the variance of each chromosome averaged across all chromosomes 1–22, and chromosome X. Low and high MKV are shown in white and green respectively (n = 3 simulations for every combination of parameters). (**C**) Population growth under each model, varying P_misseg_ and S. P_misseg_∈ [0.001, 0.022, 0.050] translate to about 0.046, 1, and 2.3 mis-segregations per division respectively for diploid cells. Top dashed line represents the population at (re-)initiation (4500 cells). Bottom dashed line represents the steady state population in selection-null conditions. (**D**) Dynamics of the average ploidy (total # chromosome arms / 46) of a population while varying P_misseg_ and S. (**E**) Dynamics of ploidy under each model for diploid and tetraploid founding populations. P_misseg_∈ [0.01, 0.02] translate to about 0.46 and 0.92 mis-segregations for diploid cells and 0.92 and 1.84 mis-segregations for tetraploid cells. (**F**) Fitness (F^S^) over time for diploid and tetraploid founding populations evolved under each model. (**G**) Karyotype diversity dynamics for diploid and tetraploid founding populations. MKV is normalized to the mean ploidy of the population at each time step. Plotted lines in C-G are local regressions of n = 3 simulations.

**Figure 2—figure supplement 2.**
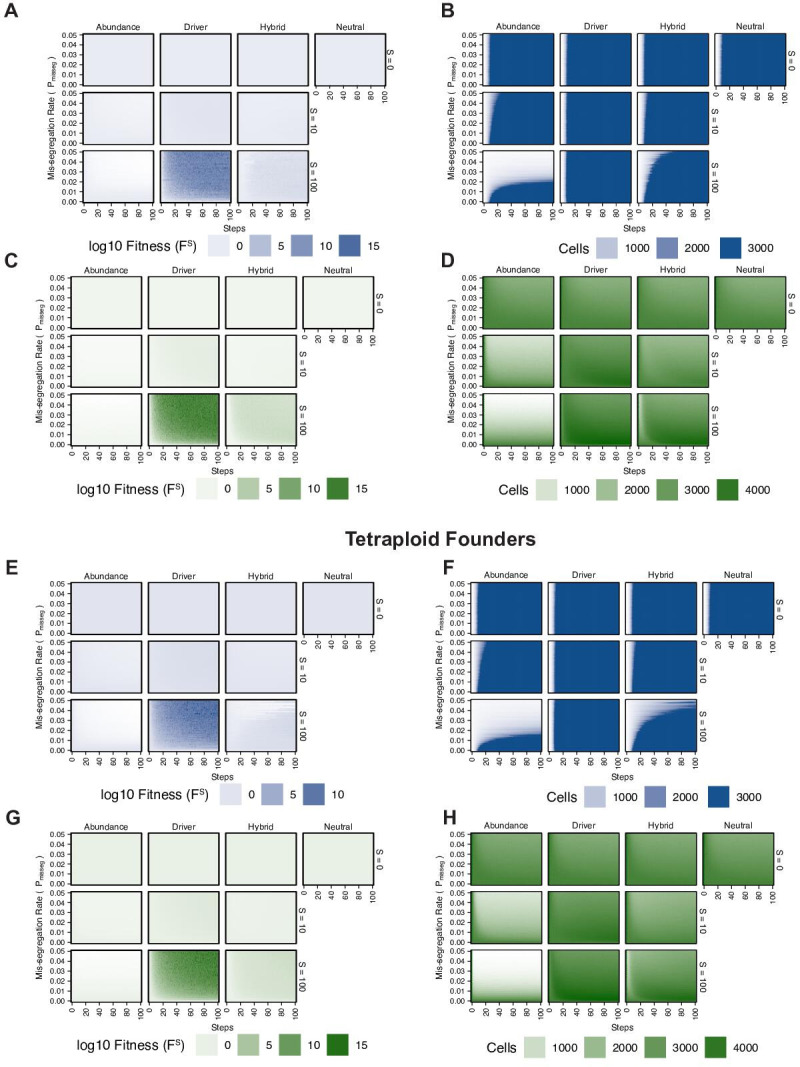
Fitness of diploid and tetraploid CIN +populations. (**A**) Fitness landscape of simulations founded by diploid cells under exponential pseudo-Moran growth dynamics. (**B**) Size of simulated populations founded by diploid cells under exponential pseudo-Moran growth dynamics. (**C**) Fitness landscape of simulations founded by diploid cells under constant Wright-Fisher growth dynamics. (**D**) Size of simulated populations founded by diploid cells under constant Wright-Fisher growth dynamics. (**E**) Fitness landscape of simulations founded by tetraploid cells under exponential pseudo-Moran growth dynamics. (**F**) Size of simulated populations founded by tetraploid cells under exponential pseudo-Moran growth dynamics. (**G**) Fitness landscape of simulations founded by tetraploid cells under constant Wright-Fisher growth dynamics. (**H**) Size of simulated populations founded by tetraploid cells under constant Wright-Fisher growth dynamics.

High levels of selection against aneuploid cells are expected to impede cell growth. To visualize this, we quantified the population of viable cells with distinct models (Figure 2C). As expected with the Abundance model at S = 10 and S = 100, cells proliferated more slowly with higher rates of mis-segregation. By contrast, the Driver model saw no growth defect as they favored specific aneuploid states that are easily reached with missegregation. As before, the Hybrid model, is intermediate, and findings are not impacted by pseudo-Moran or Wright-Fisher restrictions on cell number (Figure 2—figure supplement 1C).

To further assess model dynamics, we examined time-course of average cellular ploidy—the number of chromosomes divided by 23. In many cases, the mean ploidy of the populations tend to increase over time (Figure 2D, Figure 2—figure supplement 1D), particularly in the absence of selection (S = 0; top). This is likely due to a higher permissiveness to chromosome gains than losses in our model (since cells ‘die’ with nullisomy or any chromosome >6, the optimum is 3.0). With selection (S = 10; S = 100 rows), the models diverge. In the abundance model, populations remain near diploid. With the Driver model, the average ploidy increases more rapidly due to favoring aneuploidy states that favor high oncogenes and low tumor suppressors, consistent with previous computational models built on chromosome-specific driver densities (Davoli et al., 2013; Laughney et al., 2015). Under the Hybrid model, ploidy increases modestly. Similar effects are seen with the constant-population Wright-Fisher model (Figure 2—figure supplement 1D). In sum, selection and mis-segregation cooperate to shape the aneuploid karyotypes diversity, cell proliferation and average ploidy in a population of cells, or a human tumor. Further, sampling karyotypes in a cell population does not allow direct determination of mis-segregation rates, as their diversity is influenced by other factors such as selective pressure, selection modality, and time.

In some tumors, genome doubling occurs early in tumor initiation relative to other copy number changes (Bielski et al., 2018; Gerstung et al., 2020). Genome doubling is accomplished, for example, by endoreduplication, by failed cytokinesis, or by cell-cell fusion. Genome doubling buffers against loss of chromosomes and thereby favors aneuploidy. To determine how genome doubling impacts evolution in our model, we compared diploid and tetraploid founders (Figure 2E–G). Both diploids and tetraploids tend to converge toward the near-triploid state (ploidy ~3), as observed in many human cancers (Carter et al., 2012), although this is restrained to a degree with the Abundance and Hybrid models. Compared with diploid cells, tetraploidy buffered against the negative effects of cellular fitness in the Abundance model, despite generating similar levels of diversity over time (Figure 2F and G)— this is more pronounced when comparing P_misseg_ = 0.1 in tetraploids versus P_misseg_ = 0.2 in diploids to match the number of chromosome mis-segregations per division. This is consistent with the idea that tetraploidy serves as an intermediate enabling a near-triploid karyotype that is common in many cancers (Bielski et al., 2018; López et al., 2020). By contrast, in the Driver model, tetraploidy did not provide a selective advantage to high-CIN tumors (Figure 2F). Similar fitness, karyotype diversities, and ploidy increases were obtained with a Wright-Fisher model of population growth (Figure 2—figure supplement 1E-G, Figure 2—figure supplement 2).

Taken together, the agent-based model recapitulates expected key aspects of tumor evolution, lending credence to our model. Further, they illustrate the difficulty of inferring mis-segregation rates directly from assessing variation in karyotypes in human cancer. Nevertheless, this model provides a framework to incorporate selection to measure CIN through quantitative inference from the observed karyotypes, as we will demonstrate.

### Long-term karyotype diversity depends profoundly on selection modality

Some current measures of CIN are derived from karyotype diversity in the population. Yet, our model suggests that selection pressure will profoundly shape this diversity. To further understand the nature of karyotype diversity under selection, we evaluated their long-term dynamics, whether they exhibit clonality, and whether populations simulated under each model converge on a common karyotype.

**Figure 3.**
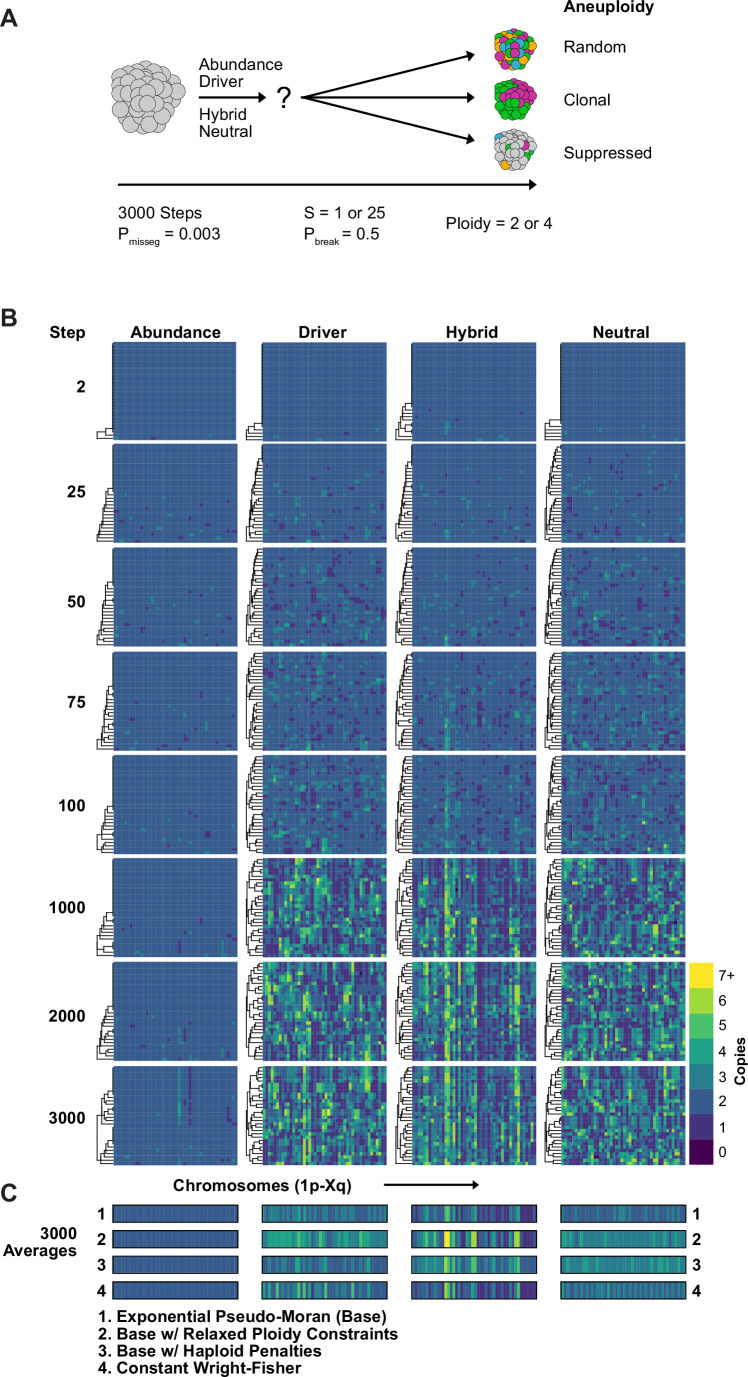
Karyotype diversity depends profoundly on selection modality. (**A**) Simulation scheme to assess long-term dynamics of karyotype evolution and karyotype convergence. (**B**) Heatmaps depicting the chromosome copy number profiles of a subset (n = 30 out of 300 sampled cells) of the simulated population with early CIN over time under each model of karyotype selection. (**C**) Average heatmaps (lower) show the average copy number across the 5 replicates for (1) the Exponential Psuedo-Moran (Base), (2) the base model with the upper copy number limit set to 10, (3) the base model that invokes a F_M_ x 0.1 penalty for any cell with a haploid chromosome, (4) and the Constant Population-Size Wright-Fisher model. P_misseg_ = 0.003; S = 25 (except Neutral model; S = 0); ploidy = 2.

We simulated diploid and tetraploid populations for 3000 time steps at a fixed mis-segregation rate, in an experimentally reported range, allowing for fragmentation of chromosome arms (P_misseg_ = 0.003, P_break_ = 0.5) (Bakhoum et al., 2009; Bolhaqueiro et al., 2019; Weaver et al., 2007) and S ∈ [1,25] (Figure 3A). We visualized copy-number heatmaps indicating karyotypes of sampled cells from the population. As expected, population diversity is limited under the Abundance model (Figure 3B). Even after 3000 time steps, only a small number of unique alterations and sub-clonal alterations ( + 13 p/–15 p/–22 p) existed, likely passenger alterations as they offer no fitness advantage in this model. Moreover, the karyotype average of 1500 cells across five replicates resembled a diploid karyotype (Figure 3C, row 1), indicating that the Abundance model provides stabilizing selection around the euploid karyotype. In fact, populations simulated under this model with elevated selection (S = 25) quickly reach a low, steady-state level of karyotype diversity and fitness while those with the unmodified selection values (S = 1) take a longer time to reach this steady-state and have similar levels of karyotype diversity and fitness as the other models (Figure 3—figure supplement 1). To identify any contingencies that may affect these associations, we performed the same simulation using several variants of our model. We found this steady state to be consistent for tetraploid cells as well as when we eased the upper ploidy constraint from nc _c_ = 6 to an extreme nc _c_ = 10, when we imposed a severe, 90% fitness reduction for all cells with a haploidy, and when we simulated populations under the Wright-Fisher model (Figure 3C, rows 2–4).

**Figure 3—figure supplement 1.**
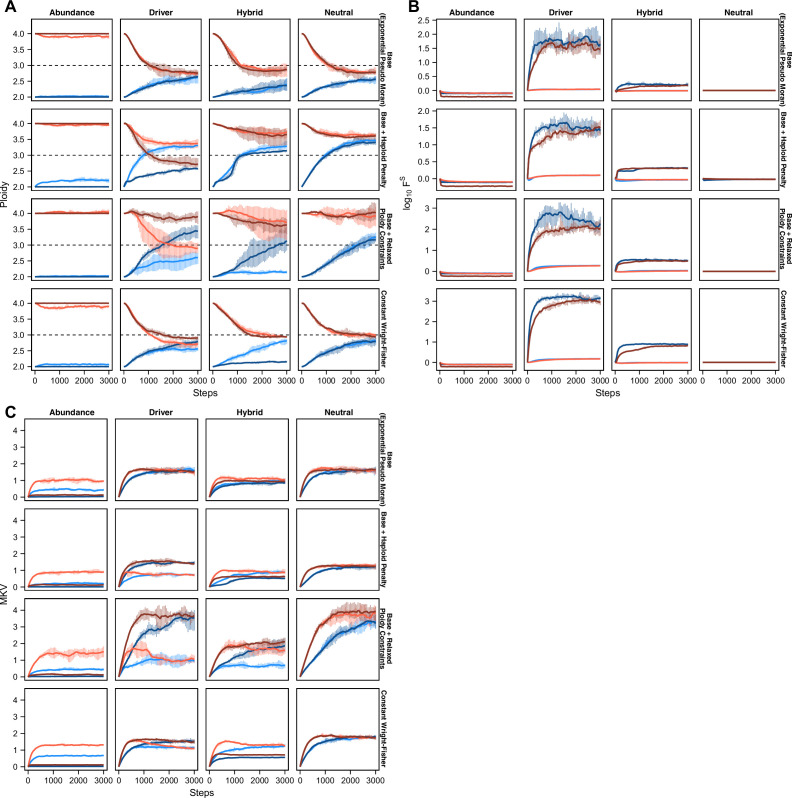
Modeled population measures tracked over time. (**A**) Average population ploidy over time for each selection model within each model variation. Data represent the mean and range (vertical lines) across five replicates for every 50 time steps in diploid populations with low selective pressure (light red) and high selective pressure (dark red) and tetraploid populations with low selective pressure (light blue) and high selective pressure (dark blue). (**B**) Average population fitness (log10) over time for each selection model within each model variation. Data represent the mean and range (vertical lines) across five replicates for every 50 time steps in diploid populations with low selective pressure (light red) and high selective pressure (dark red) and tetraploid populations with low selective pressure (light blue) and high selective pressure (dark blue). (**C**) Mean karyotype variance over time for each selection model within each model variation. Data represent the mean and range (vertical lines) across five replicates for every 50 time steps in diploid populations with low selective pressure (light red) and high selective pressure (dark red) and tetraploid populations with low selective pressure (light blue) and high selective pressure (dark blue).

The Driver Density and Hybrid models generate much more diversity (Figure 3B) but nevertheless converge by 3,000 timesteps (Figure 3—figure supplement 1). Without selection (neutral model), there is high diversity and no convergence over time. Taken together, these demonstrate a high dependence on the model of selection. However, the models are not highly dependent on ploidy constraints, haploid penalties, or on selection of Pseudo-Moran or Wright-Fisher restriction of cell numbers. Taken together, long-term populations are strongly shaped by the model of karyotype selection for a given P_misseg_, but relatively insensitive to other particular features of the model. This justifies our approach henceforth of varying only the selection model, the degree of selection (S), and P_misseg_ to infer parameters from data via phylogenetic topology and Bayesian inference.

### Topological features of simulated phylogenies delineate CIN rate and karyotype selection

Given a model capable of recapitulating diversity and selective pressures, next we wish to infer P_misseg_ as a measure of CIN from an observed population of cells. Phylogenetic trees provide insights into evolutionary processes of genetic diversification and selection. Moreover, the topology of the phylogenetic tree has been used as a quantitative measure of the underlying evolutionary processes (Colijn and Plazzotta, 2018; Dayarian and Shraiman, 2014; Manceau et al., 2015; Neher et al., 2014; Scott et al., 2020).

**Figure 4.**
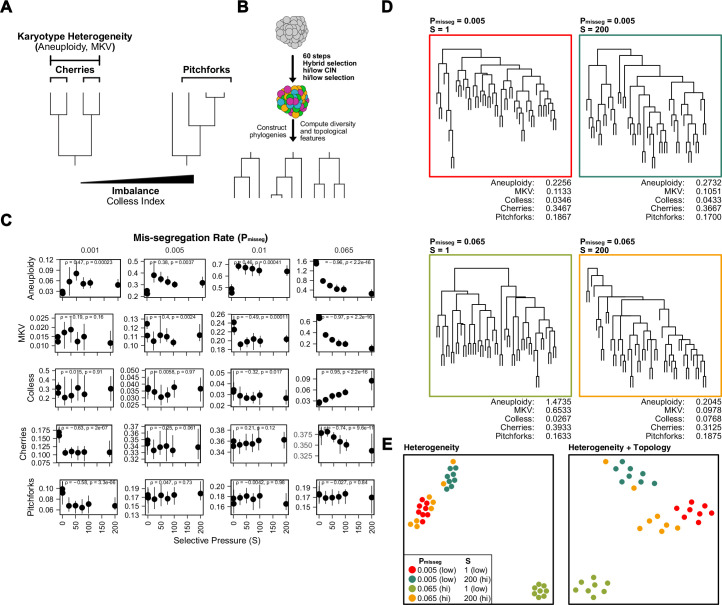
Topological features of simulated phylogenies delineate CIN rate and karyotype selection. (**A**) Quantifiable features of karyotypically diverse populations. Heterogeneity between and within karyotypes is described by MKV and aneuploidy (inter- and intra-karyotype variance, see Materials and methods). We also quantify discrete topological features of phylogenetic trees, such as cherries (tip pairs) and pitchforks (3-tip groups), and a whole-tree measure of imbalance (or asymmetry), the Colless index. (**B**) Scheme to test how CIN and selection influence the phylogenetic topology of simulated populations. (**C**) Computed heterogeneity (aneuploidy and MKV) and topology (Colless index, cherries, pitchforks) summary statistics under varying P_misseg_ and S values. MKV is normalized to the average ploidy of the population. Topological measures are normalized to population size. Spearman rank correlation coefficients (r) and p-values are displayed (n = 8 simulations). (**D**) Representative phylogenies for each hi/low CIN, hi/low selection parameter combination and their computed summary statistics. Each phylogeny represents n = 50 out of 300 cells for each simulation. (**E**) Dimensionality reduction of all simulations for each hi/low CIN, hi/low selection parameter combination using measures of karyotype heterogeneity only (left; MKV and aneuploidy) or measures of karyotype heterogeneity and phylogenetic topology (right; MKV, aneuploidy, Colless index, cherries, and pitchforks).

Here, chromosome mis-segregation gives rise to karyotype heterogeneity, and the population of cells is then shaped by selection. To evaluate this, we use chromosome copy number-based phylogenetic reconstruction, since mutation rates are not high enough in tumors to reliably infer cellular relationships, particularly with low-copy sequencing. Once phylogenies are reconstructed from simulated and experimental populations, the topological features phylogenies can be compared. These features include ‘cherries’—two tips that share a direct ancestor—and ‘pitchforks—a clade with three tips (Figure 4A). Additionally, we considered a broader metric of topology, the Colless index, which measures the imbalance or asymmetry of the entire tree. To understand how these measures are affected by selection in simulated populations, we reconstructed phylogenies from 300 random cells from each population simulated with a range of selective pressures taken at 60 time steps (~30 divisions under Hybrid selection; Figure 4B). As seen previously, aneuploidy and mean karyotypic variance (MKV) decrease with selective pressure, a trend that is robust at high mis-segregation rates (Figure 4C). By contrast, Colless indices increase with mis-segregation rates and selective pressures, as the resulting variation and selection generate phylogenetic asymmetry. Accordingly, this imbalance is apparent in phylogenetic reconstructions of simulated populations (Figure 4D). Cherries, by contrast, decrease with selection due to selection against many aneuploidies (Figure 4C). Pitchforks seemed less informative. Therefore, we tentatively selected 4 phylogenetic parameters that can retain information about chromosome missegregation—aneuploidy, MKV, Colless, and Cherries.

To characterize how well the four measures retain information about the simulation parameters, we performed dimensionality reduction with measures of karyotype heterogeneity alone (MKV and aneuploidy) alone and adding Colless and cherries—measures of phylogenetic topology (Figure 4E). This analysis indicates that when considering heterogeneity alone simulations performed under high CIN/high selection (yellow) and low CIN/low selection (red) associate closely, meaning these measures of heterogeneity are not sufficient to distinguish these disparate conditions (Figure 4E, left). These similarities arise because high selection can mask the heterogeneity expected from high CIN. By contrast, combining measures of heterogeneity with those of phylogenetic topology can discriminate between simulations with disparate levels of CIN and selection (Figure 4E, right). This provides further evidence that measures of heterogeneity alone are not sufficient to infer CIN due to the confounding effects of selection, particularly when the nature of selection is unclear or can vary. Together these results indicate that phylogenetic topology preserves information about underlying levels of selective pressure and rates of chromosome mis-segregation. Further, phylogenetic topology of single-cell populations may be a suitable way to correct for selective pressure when estimating the rate of chromosome mis-segregation from measures of karyotype diversity.

### Experimental chromosome mis-segregation measured by Bayesian inference

**Figure 5.**
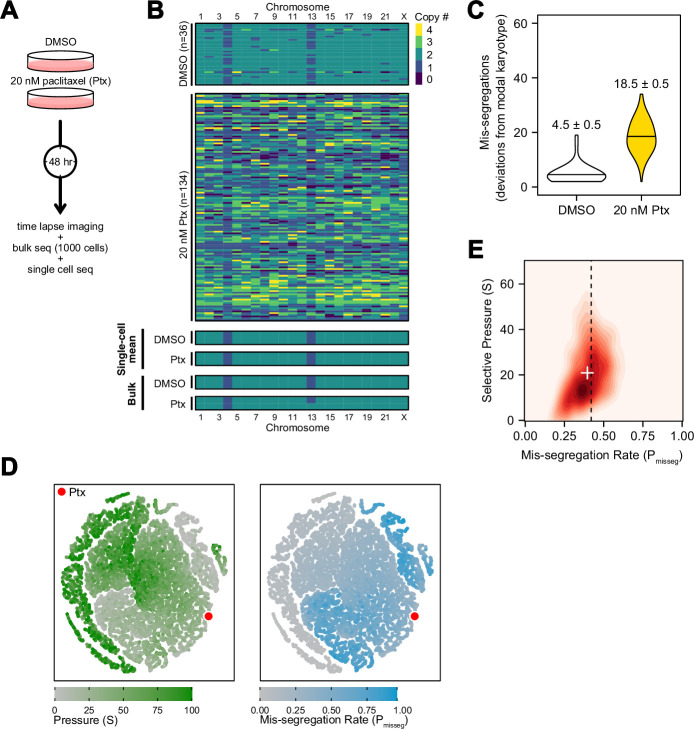
Experimental chromosome mis-segregation measured by Bayesian inference experimental scheme. (**A**) Cal51 cells were treated with either DMSO or 20 nM paclitaxel for 48 hr prior to further analysis by time lapse imaging, bulk DNA sequencing, and scDNAseq. (**B**) Heatmaps showing copy number profiles derived from scDNAseq data, single-cell copy number averages, and bulk DNA sequencing. (**C**) Observed mis-segregations calculated as the absolute sum of deviations from the observed modal karyotype of the control. (**D**) Dimensionality reduction analysis of population summary statistics (aneuploidy, MKV, Colless index, cherries) from the first three time steps of all simulations performed under the Hybrid model. (**E**) 2D density plot showing joint posterior distributions from ABC analysis using population summary statistics computed from the paclitaxel-treated cells using the following priors and parameters: Growth Model = ‘exponential pseudo-Moran’, Selection Model = ‘Hybrid, initial ploidy = 2, 2 time steps, S ∈[0, 2… 100], P_misseg_∈[0, 0.005… 1.00] and a tolerance threshold of 0.05 to reject dissimilar simulation results. (see Materials and Methods). Vertical dashed line represents the experimentally observed mis-segregation rate. White + represents the mean of inferred values.

To experimentally validate quantitative measures of CIN, we generated a high rate of chromosome mis-segregation with a clinically relevant concentration of paclitaxel (Taxol) over 48 hr (Figure 5A). We treated CAL51 breast cancer cells with either a DMSO control or 20 nM paclitaxel, which generates widespread aneuploidy due to chromosome mis-segregation on multipolar mitotic spindles (Zasadil et al., 2014), verified in this experiment (Figure 5—figure supplement 1A). At 48 hr cells will have undergone 1–2 mitoses and, consistent with abnormal chromosome segregation, we observe broadened DNA content distributions by flow cytometry (Figure 5—figure supplement 1B). Using low-coverage scDNAseq data, we characterized the karyotypes of 36 DMSO- and 134 paclitaxel-treated cells. As expected, virtually all cells had extensive aneuploidy after paclitaxel, in contrast with low variance in the control (Figure 5B). Additionally, the mean of the resultant aneuploid karyotypes for each chromosome still resembled those of bulk-sequenced cells, highlighting that bulk-sequencing is an ensemble average, and does not detect variation in population aneuploidy, particularly with balanced mis-segregation events (Figure 5B, single-cell mean and bulk). In quantifying the absolute deviation from the modal control karyotype in each cell, and assuming a single mitosis, cells exposed to 20 nM paclitaxel mis-segregate 18.5 ± 0.5—a P_misseg_ of ~0.42 considering the control’s sub-diploid modal karyotype (Figure 5C). The majority of these appeared to be whole-chromosome mis-segregations (Figure 5—figure supplement 2).

**Figure 5—figure supplement 1.**
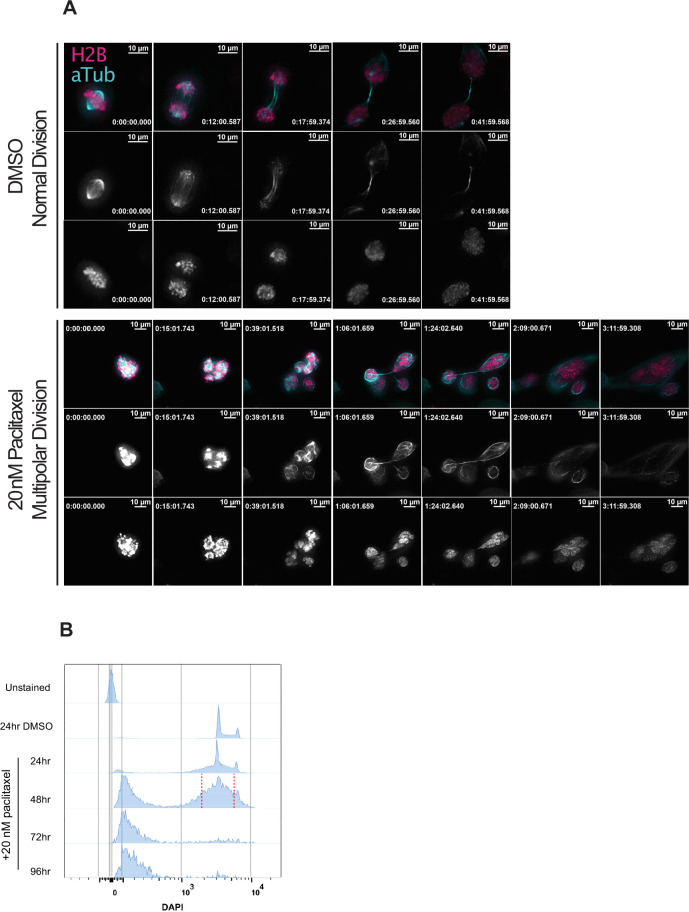
Induction of extensive chromosome mis-segregation via paclitaxel. (**A**) Immunofluorescence time lapse montage of control Cal51 cells undergoing normal mitosis (top) and paclitaxel-treated treated cells undergoing a multipolar anaphase (middle) and partial cytokinesis failure (bottom). (**B**) Cell cycle profiles from flow cytometric analysis of Cal51 cells treated with either DMSO (72 hr) or 20 nM paclitaxel for 24, 48, or 72 hr. For FACS, cells treated for 48 hr were sorted into individual wells of 96-well plates. Sorting gate is shown by the red, dashed line.

**Figure 5—figure supplement 2.**
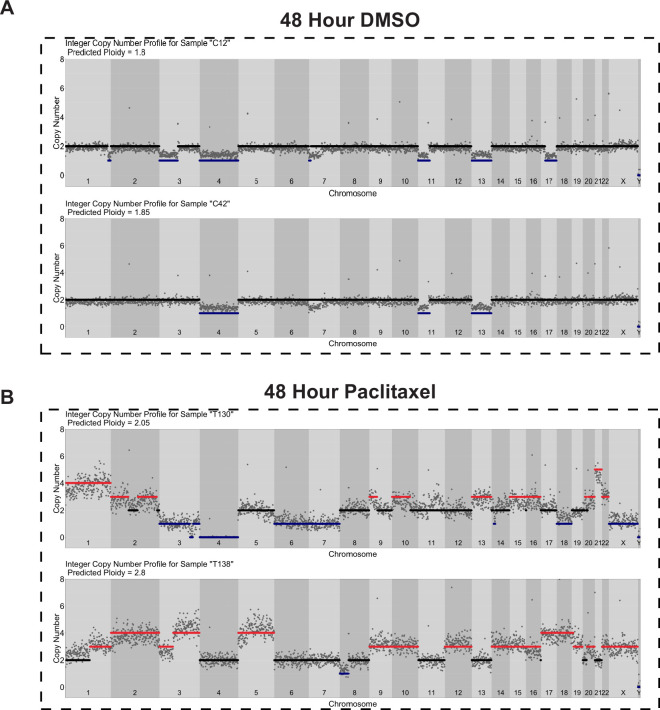
Copy number profiles of DMSO- and paclitaxel-treated Cal51 cells. Single-cell copy number profiles for single (**A**) DMSO- and (**B**) paclitaxel-treated cells. A total of 500 Kb genomic bins and DNA content from FACS were used for copy number calculations (see Materials and methods).

**Figure 5—figure supplement 3.**
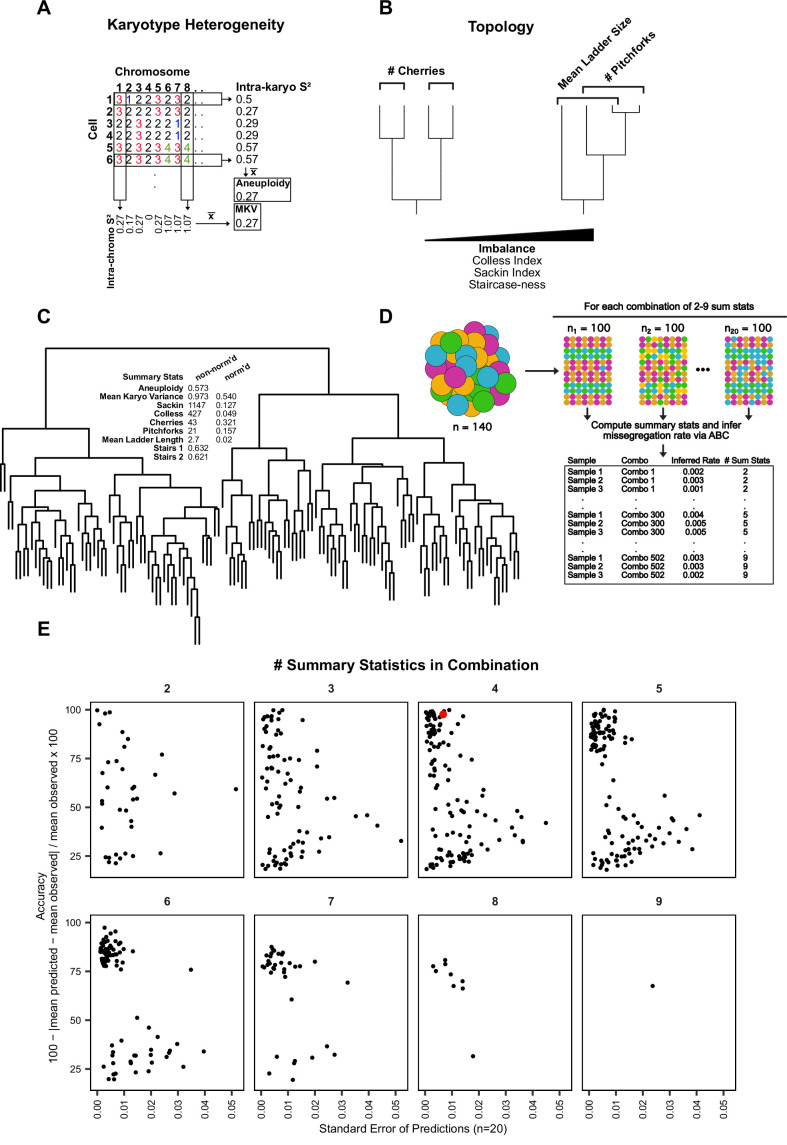
Summary statistic optimization for ABC. (**A**) Schematic showing calculation of aneuploidy and MKV. (**B**) Examples of phylogenetic topology metrics. (**C**) Phylogenetic reconstruction of a population of Cal51 cells treated with 20 nM paclitaxel for 48 hr and associated heterogeneity and topology metrics. Normalized and non-normalized summary statistics are displayed (see Materials and methods). (**D**) Analytical scheme to identify most accurate and least variable combinations of heterogeneity and topology metrics. For each combination of 2–9 metrics, we iteratively re-sampled and remeasured the rate of mis-segregation in 100 random cells, three times, from our original dataset of paclitaxel-treated Cal51 cells. The red data point denotes our chosen combination for future analyses—average aneuploidy, MKV, Colless Index, and Cherries. This combination both limits redundant measures (i.e. Colless and Sackin indices) and contains both heterogeneity and topology metrics. (**E**) Percent accuracy and standard error of the mean for three sampled measurements of 100 paclitaxel-treated cells from the original population, repeated for each combination of heterogeneity and topology measures.

**Figure 5—figure supplement 4.**
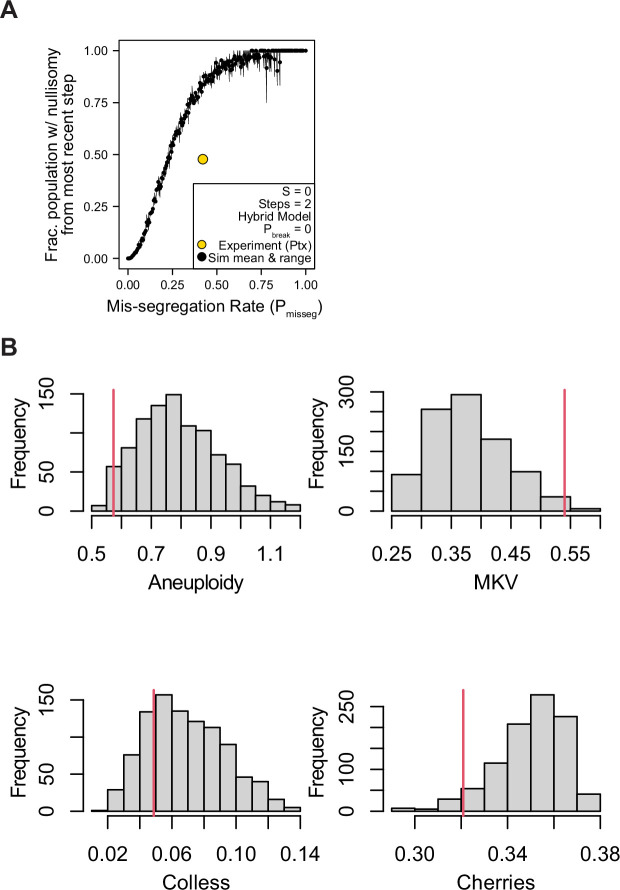
Nullisomy and posterior predictive checks of summary statistics from paclitaxel-treated Cal51 cells. (**A**) Observed incidence of nullisomy in paclitaxel-treated cells plotted against the observed mis-segregation rate (P_misseg,true_ = 18.5/44 = 0.42) overlaid on simulated data from the second time step (2 generations) under the Hybrid model with S = 0 and P_break_ = 0 (n = 3 simulations). (**B**) Posterior distributions of summary statistics from accepted simulations most similar to the paclitaxel-treated Cal51 cells (threshold = 0.05). The red line indicates the observed statistic in paclitaxel-treated cells. Colless index and cherry count is normalized to population size. MKV is normalized to the average ploidy of the population.

**Figure 5—figure supplement 5.**
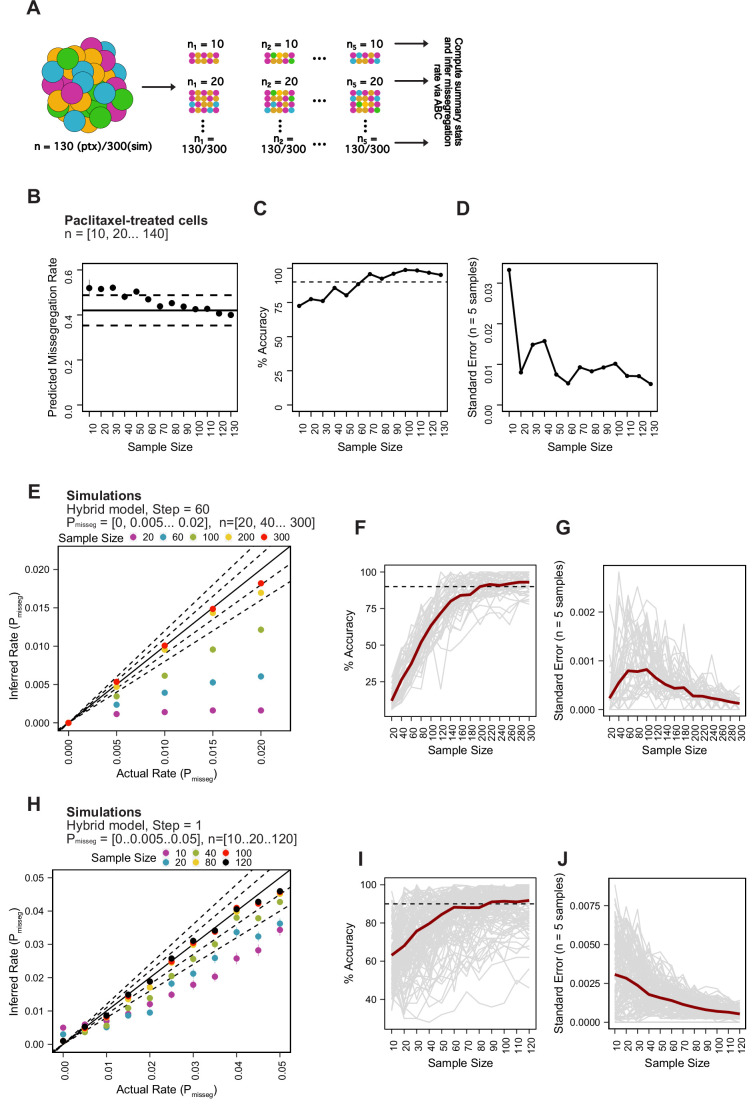
Minimum sampling of karyotype heterogeneity. (**A**) Analytical scheme to optimize the number of cells to sample for measuring mis-segregation rates from karyotype heterogeneity. We iteratively re-sampled and remeasured the rate of mis-segregation for a range of sample sizes (n = 5 random samples). (**B**) Predicted mis-segregation rates over a range of sample sizes (n = 5 samples). Points and error bars are the mean ± standard error. Black solid line denotes the mean observed rate of mis-segregation induced by 20 nM paclitaxel. Black dashed lines are half the standard deviation of observed mis-segregation rates per cell. (**C**) Mean percent accuracy of ABC-inferred rates of mis-segregation due to paclitaxel taken from each set of five random samples using the observed rate of mis-segregation as the ‘true value’. Calculated as $Mean\;\%\;accuracy=100-\left(\frac{true-mean\;inferred}{true}\times 100\right)$. Dashed lines represent 90% accuracy. (**D**) Standard error of ABC-inferred rates of mis-segregation for each set of random samples from paclitaxel-treated cells. (**E**) ABC-inferred mis-segregation rates by sample size from simulations with known parameters (n = 5 samples). Points represent mean ± standard error across 5 samples for each of 11 selective pressure (S) values. Solid line represents a perfect correlation. Inner dashed line represent ±10% margin. Outer dashed line represents ±20% margin. Simulation parameters: P_misseg_∈ [0, 0.005… 0.02], time steps = 60, Selection Model = ‘Hybrid’, Growth Model = ‘exponential pseudo-Moran’, S = [0, 10... 100], and a tolerance threshold of 0.05. (**F**) Mean percent accuracy of ABC-inferred rates of mis-segregation in simulations (parameters in **E**) taken at various sample sizes. Gray lines represent the mean percent accuracy of five random samples for each sample size for the same simulated population (n = 55 simulations). The dashed line represents 90% accuracy. Calculated as described above but taking the known simulation parameter as the ‘true’ value. (**G**) Standard error of ABC-inferred rates of mis-segregation in simulations (parameters in **E**) taken at various sample sizes. Gray lines represent the standard error of five random samples for each sample size for the same simulated population (n = 55 simulations). (**H**) ABC-inferred mis-segregation rates by sample size from simulations with known parameters (n = 5 samples). Points represent mean ± standard error across 5 samples for each of 11 selective pressure (S) values. Solid line represents a perfect correlation. Inner dashed line represent ±10% margin. Outer dashed line represents ±20% margin. ABC was performed with the following parameters and priors: P_misseg_∈[0, 0.005… 0.05], time steps = 1, Selection Model = ‘Hybrid’, Growth Model = ‘exponential pseudo-Moran’, S ∈ [0, 10… 100], and a tolerance threshold of 0.05. (**I**) Mean percent accuracy of ABC-inferred rates of mis-segregation in simulations (parameters in **H**) taken at various sample sizes. Gray lines represent the mean percent accuracy of five random samples for each sample size for the same simulated population (n = 121 simulations). The dashed line represents 90% accuracy. (**J**) Standard error of ABC-inferred rates of mis-segregation in simulations (parameters in **H**) taken at various sample sizes. Gray lines represent the standard error of five random samples for each sample size for the same simulated population (n = 121 simulations). Note: Red lines in **F**, **G**, **I**, and **J** represent the median.

In this instance, we were able to estimate mis-segregation rate by calculating absolute deviation from the modal karyotype after a single aberrant cell division. However, such an analysis would not be possible for long-term experiments, or real tumors, where new aneuploid cells may be subject to selection. Accordingly, we sought to infer the parameters of this experiment—the mis-segregation rate of 18.5 chromosomes per division and low selection—using only measures of aneuploidy, variance, and phylogenetic topology. To display this, we used dimensionality reduction to ensure that observed measures from the paclitaxel-treated Cal51 population fell within the space of those observed from simulated populations over 2 steps under the Hybrid model. The experimental data mapped to those from simulations using high mis-segregation rates and relatively low selection (red point, Figure 5D). However, this comparison does not provide a quantitative measure of CIN. Instead, parameter inference via approximate Bayesian computation (ABC) is well suited for this purpose.

By deriving phylogeny metrics from simulated populations under a wide-range of distributions of evolutionary parameters, ABC identifies evolutionary parameters most consistent with the data—the posterior probability distribution. We used ABC with simulated data to infer the chromosome mis-segregation rate and selective pressure in the paclitaxel-treated cells (Csilléry et al., 2012). Importantly, this data has directly observed rates of mis-segregation, which provide a gold standard benchmark to optimize ABC inference.

One key aspect of ABC is the selection of optimal phylogenetic summary statistics. A small number of summary statistics is optimal and larger numbers impair the model (Csilléry et al., 2012). To address this, a common approach is to identify a small set of summary statistics that achieve the best inference. Here, we used the experimentally observed mis-segregation rate as a benchmark to optimally select a panel of measures for parameter inference (Figure 5—figure supplement 3) and selected the following four metrics to use concurrently in our ABC analysis: mean aneuploidy, MKV, the Colless index (a phylogenetic balance index) and number of cherries (normalized to population size). In doing so, this analysis inferred a chromosome mis-segregation rate of 0.396 ± 0.003 (or 17.4 ± 0.1 chromosomes; mean ± SE), which compares favorably with the experimentally observed rate of 18.5 ± 0.5 (Figure 5E; dashed line represents experimental rate, white ‘+’ the inferred rate). The distribution of accepted values for selection was skewed toward lower pressure (21 ± 0.4; mean ± SE), meaning that karyotype selection had little bearing on the result at this time point, consistent with the absence of selection in a 48-hr experiment.

Interestingly, the incidence of nullisomy in the simulated population was higher than in the paclitaxel-treated populations at the observed mis-segregation rate (Figure 5—figure supplement 4A). This could be due to spindle pole clustering, a recovery mechanism often seen in paclitaxel-treated cells that causes non-random chromosome mis-segregations. A posterior predictive check of the summary statistics demonstrates how each contributes to the inference of CIN rate (Figure 5—figure supplement 4B). In short, this experimental case validated ABC-derived mis-segregation rate as a measure of CIN, with an experimentally determined mis-segregation rate. Importantly, prior estimations of mis-segregation rate selective pressure were not required to develop this quantitative measure of CIN.

Together, these data indicate that combining simulated and observed metrics of population diversity and structure with a Bayesian framework for parameter inference is a flexible method of quantifying the evolutionary forces associated with CIN. Moreover, this method reveals the hitherto unreported potential extent of chromosome mis-segregation induced by a clinically relevant concentration of the successful chemotherapeutic paclitaxel consistent with the measured mis-segregation from non-pharmacologically induced multipolar divisions (Bollen et al., 2021).

### Minimum sampling of karyotype heterogeneity

The cost of high-throughput DNA sequencing of single cells is often cited as a limitation to clinical implementation (Evrony et al., 2021). In part, the cost can be limited by low-coverage sequencing which is sufficient to estimate the density of reads across the genome. Further, it may be possible to minimize the number of cells that are sampled to get a robust estimate of CIN, though sampling too few cells may result in inaccurate measurements. Accordingly, we determined how sampling impacts measurement of mis-segregation rates using approximate Bayesian computation. We first took five random samples from the population of paclitaxel-treated cells each at various sample sizes (Figure 5—figure supplement 5A). We then inferred the mis-segregation rate in each sample and identified the sample size that surpasses an average of 90% accuracy and a low standard error of measurement. We found that even small sample sizes can accurately infer the mis-segregation rate, in this context, with a low standard error (Figure 5—figure supplement 5B-D). A sample size of 60 cells produced the most accurate measurement at 99.5% and a standard error of 0.008 ( ± 0.35 chromosomes). We repeated this analysis using simulated data from the Hybrid selection model and a range of mis-segregation rates spanning what is observed in cancer and non-cancer cultures (P_misseg_ ≤ 0.02; see below). We again found a range of sample sizes whose inferred mis-segregation rates underestimate the known value from those simulations (n∈ [20, 40… 180]; Figure 5—figure supplement 5E,F). Across all mis-segregation rates and selective pressures, random samples of 200 cells had a median percent accuracy of 90% and median standard error of 0.0003 ( ± 0.0138 chromosomes per division). The difference in optimal sample sizes between the paclitaxel-treated population and the simulated population is notable and likely due to the presence of ‘clonal’ structures in the simulated population. While the paclitaxel treatment resulted in a uniformly high degree of aneuploidy and little evidence of karyotype selection, the simulated populations after 60 steps (~30 generations) have discrete copy number clusters that may not be captured in each random sample. To verify this, we repeated the analysis using only data from the first time step, prior to the onset of karyotype selection (Figure 5—figure supplement 5H). In this case, we found that the sample size needed to achieve a median 90% accuracy over all simulations in this context is 100 cells, at which point the standard error for P_misseg_ is 0.0068 (placing measures within ±0.31 chromosomes per division; Figure 5—figure supplement 5I, J). Thus, a larger number of cells is required in the context of long-term karyotype selection than a more acute time scale, such as we see with paclitaxel.

In conclusion, we recommend using 200 cells from a single sampled site which, at biologically relevant time scales and rates of mis-segregation, provides ≥90% accuracy. These data represent, to our knowledge, the first analysis of how sample size for single-cell sequencing affects the accuracy and measurement of chromosome mis-segregation rates.

### Inferring chromosome mis-segregation rates in tumors and organoids

**Figure 6.**
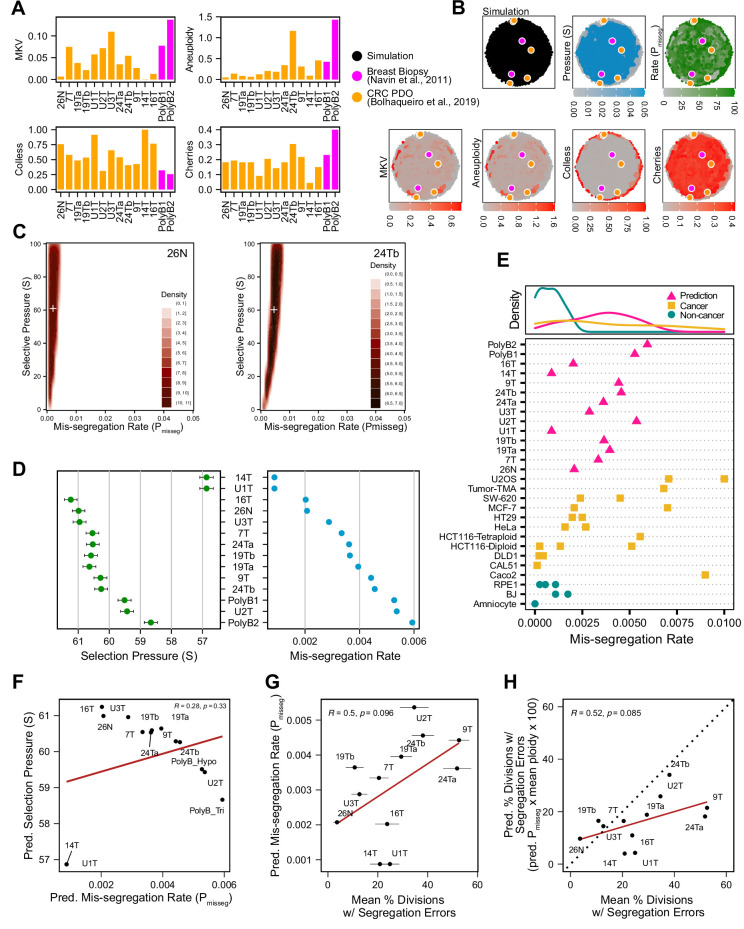
Inferring chromosome mis-segregation rates in tumors and organoids [Bibr bib8][Bibr bib52]. (**A**) Computed population summary statistics for colorectal cancer (CRC) patient-derived organoids (PDOs) and breast biopsy scDNAseq datasets from Bolhaqueiro et al., 2019 (gold) and Navin et al., 2011 (pink). (**B**) Dimensionality reduction analysis of population summary statistics showing biological observations overlaid on, and found within, the space of simulated observations. Point colors show the simulation parameters and summary statistics for all simulations using the following priors and parameters: Growth Model = ‘exponential pseudo-Moran’, Selection Model = ‘Abundance’, initial ploidy = 2, time steps ∈[40, 41… 80], S ∈[0,2… 100], P_misseg_∈[0,0.001… 0.050] and a tolerance threshold of 0.05 to reject dissimilar simulation results. (see Materials and Methods). (**C**) 2D density plots showing joint posterior distributions of P_misseg_ and S values from the approximate Bayesian computation analysis of samples 26 N (left) and 24Tb (right) from Bolhaqueiro et al., 2019. White + represents the mean of inferred values. (**D**) Inferred selective pressures and mis-segregation rates from each scDNAseq dataset (mean and SEM of accepted values). (**E**) Predicted mis-segregation rates in CRC PDOs and a breast biopsy plotted with approximated mis-segregation rates observed in cancer (blue triangle) and non-cancer (red circle) models (primarily cell lines) from previous studies (Table 5; see Materials and methods). The predicted mis-segregation rates in these cancer-derived samples fall within those observed in cancer cell lines and above those of non-cancer cell lines. (**F**) Pearson correlation of predicted mis-segregation rates and predicted selective pressures in CRC PDOs from Bolhaqueiro et al., 2019. (**G**) Pearson correlation of predicted mis-segregation rates and the incidence of observed segregation errors in CRC PDOs from Bolhaqueiro et al., 2019. Error bars represent SEM values. (**H**) Pearson correlation of observed incidence of segregation errors in CRC PDOs from Bolhaqueiro et al., 2019 to the ploidy-corrected prediction of the observed incidence of segregation errors. These values assume the involvement of 1 chromosome per observed error and are calculated as the (predicted mis-segregation rate) x (mean number of chromosomes observed per cell) x 100. Dotted line = 1:1 reference.

To determine if this framework is clinically applicable, we employed previously published scDNAseq datasets derived from tumor samples and patient-derived organoids (PDO) (Bolhaqueiro et al., 2019; Navin et al., 2011). Importantly, the data from Bolhaqueiro et al. include sample-matched live cell imaging data in colorectal cancer PDOs, with direct observation of chromosome mis-segregation events to compare with inferred measures. We established our panel of measurements on these populations (Figure 6A) and used these to tune the prior distribution of time steps and the rejection threshold for ABC. In sensitivity analysis, 20 steps or greater was sufficient to establish stable estimates of P_misseg_ and selection, S (Figure 6—figure supplement 1A-B)—we chose a window of 40–80 steps for further analysis. For rejection thresholds 0.05 and smaller, the inferred mis-segregation rates remained steady (Figure 6—figure supplement 1C). With these model parameters chosen, we evaluated the different selection models, and found that the Abundance model resulted in simulated data that best resembled experimental data, for both exponential and constant-population dynamics (Table 3). Given that the Abundance model is the most biologically relevant, we will use data simulated under this model in our prior dataset for inference.

**Figure 6—figure supplement 1.**
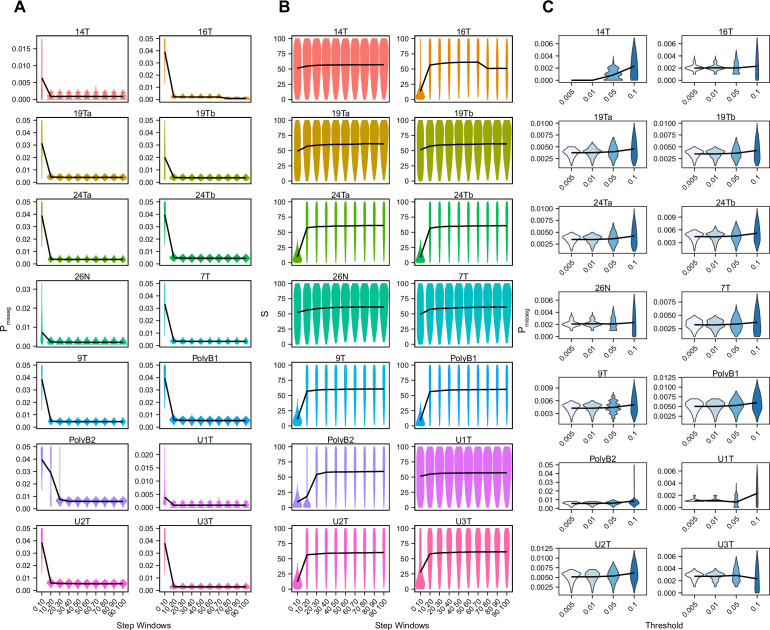
ABC-inference threshold and step-window analysis. Posterior distributions of mis-segregation rates (**A**) and selective pressure, S (**B**) inferred using ABC analysis of CRC organoids and a breast biopsy from Bolhaqueiro et al., 2019 and Navin et al., 2011 respectively using a sliding window prior distribution of time steps. ABC was performed for every interval of 10 steps between 0 and 100 using a tolerance threshold of 0.05. Schematic of analysis shown below. ABC was performed with the following parameters and priors: P_misseg_∈ [0...0.001...0.05], S ∈ [0...2...100], indicated time step window, Selection Model = ‘Abundance’, Growth Model = ‘exponential pseudo-Moran’, and a tolerance threshold of 0.05. (**C**) Posterior distributions of mis-segregation rates inferred using ABC analysis on the same samples as in A using tolerance thresholds of 0.005, 0.01, 0.05, 0.1. ABC was performed with the following parameters and priors: P_misseg_∈ [0, 0.001… 0.05], S ∈ [0, 2… 100], time steps ∈ [40, 41… 80], Selection Model = ‘Abundance’, Growth Model = ‘exponential pseudo-Moran’, and the indicated tolerance threshold.

**Figure 6—figure supplement 2.**
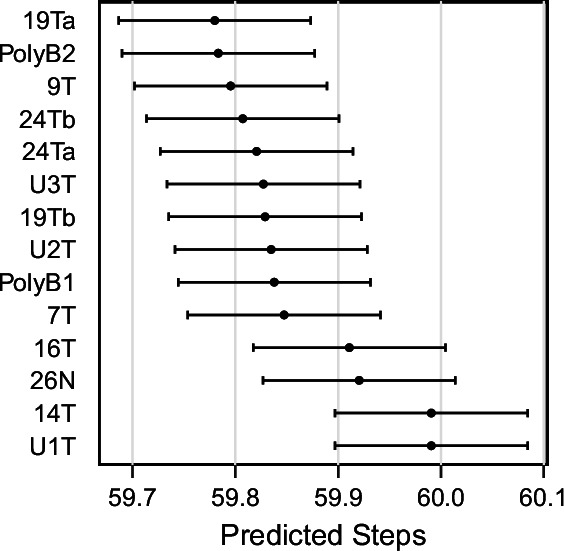
ABC-inferred step count in patient-derived samples. Mean and standard error for steps in each patient-derived sample (accompanying data in Figure 6), inferred via approximate Bayesian computation.

**Figure 6—figure supplement 3.**
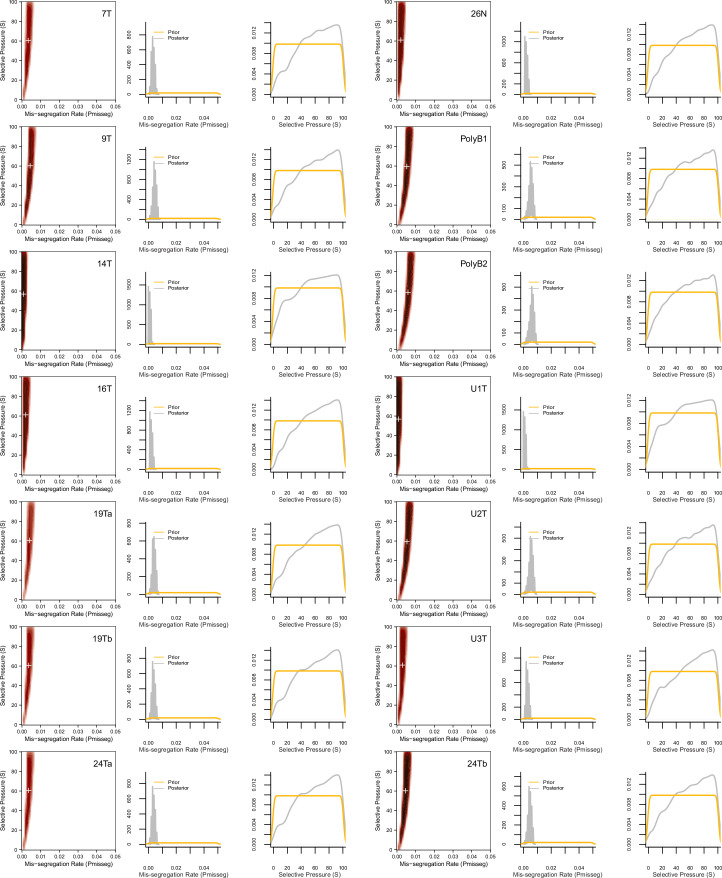
ABC-inferred mis-segregation rates and selective pressures in patient-derived samples. Joint (2D density plots) and individual (1D density plots) distributions of mis-segregation rates and selective pressures in patient-derived CRC organoids and a breast biopsy from Bolhaqueiro et al., 2019 and Navin et al., 2011 respectively (accompanying data in Figure 6). The prior (yellow) distribution represents the parameters used for simulation while the posterior (gray) distribution represents the parameters from simulations whose observed measurements were similar to the measurements taken from the patient-derived sample using a tolerance threshold of 0.05. White + signs on joint distributions represent the mean of both parameters.

**Figure 6—figure supplement 4.**
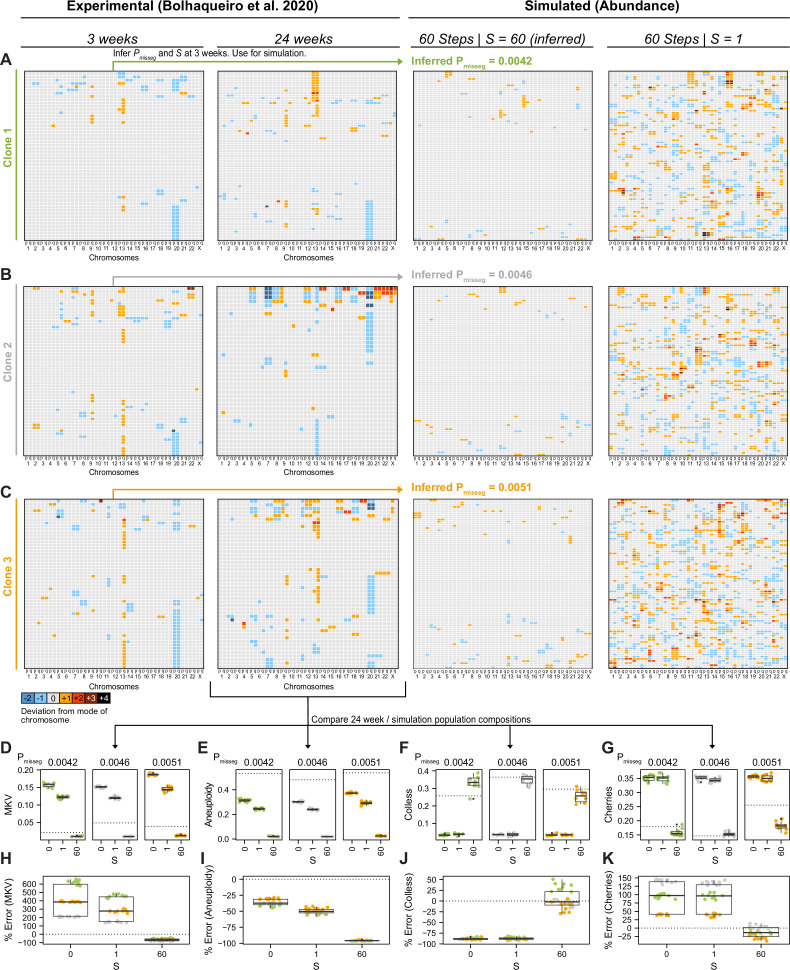
Validation of selection in longitudinally sequenced CRC organoids. (**A–C**) Copy number heatmaps showing the deviation from the mode of each chromosome derived from longitudinally sequenced clonal organoids from Bolhaqueiro et al., 2019. ABC was performed on scDNAseq data from three clones at 3 weeks of growth. The resulting inferred mis-segregation rate (P_misseg_) and selective pressure (S) were used to simulate CIN and selection in these clones over 60 time steps, at which point the composition of the populations were compared to the scDNAseq data from each of the clones at 24 weeks of growth (**D–K**). Additional simulations using S = 0 (not shown) and S = 1 were also performed. Inferred P_misseg_ values for (**A**) clone 1, (**B**) clone 2, and (**C**) clone 3 were 0.0042, 0.0046, and 0.0051 respectively. S = 60 was inferred for each clone. ABC was performed on the 3 week data with the following parameters and priors: P_misseg_∈ [0, 0.001... 0.05], S ∈ [0, 2… 100], time steps ∈ [40, 41... 80], Selection Model = ‘Abundance’, Growth Model = ‘exponential pseudo-Moran’, and a tolerance threshold of 0.05. (**D**) MKV values from n = 10 simulations per clone. Dotted line represents the MKV value observed in the scDNAseq data. (**E**) Aneuploidy values from n = 10 simulations per clone per S value. Dotted line represents the Aneuploidy value observed in the scDNAseq data. (**F**) Colless index values from n = 10 simulations per clone S value. Dotted line represents the Colless index value observed in the scDNAseq data. (**G**) Normalized cherry values from n = 10 simulations per clone S value. Dotted line represents the normalized cherry value observed in the scDNAseq data. (**H**) Percent error for MKV observations in n = 10 simulations per clone per S value. Dotted line represents 0% error. (**I**) Percent error for aneuploidy observations in n = 10 simulations per clone per S value. Dotted line represents 0% error. (**J**) Percent error for Colless observations in n = 10 simulations per clone per S value. Dotted line represents 0% error. (**K**) Percent error for normalized cherry observations in n = 10 simulations per clone per S value. Dotted line represents 0% error.

**Figure 6—figure supplement 5.**
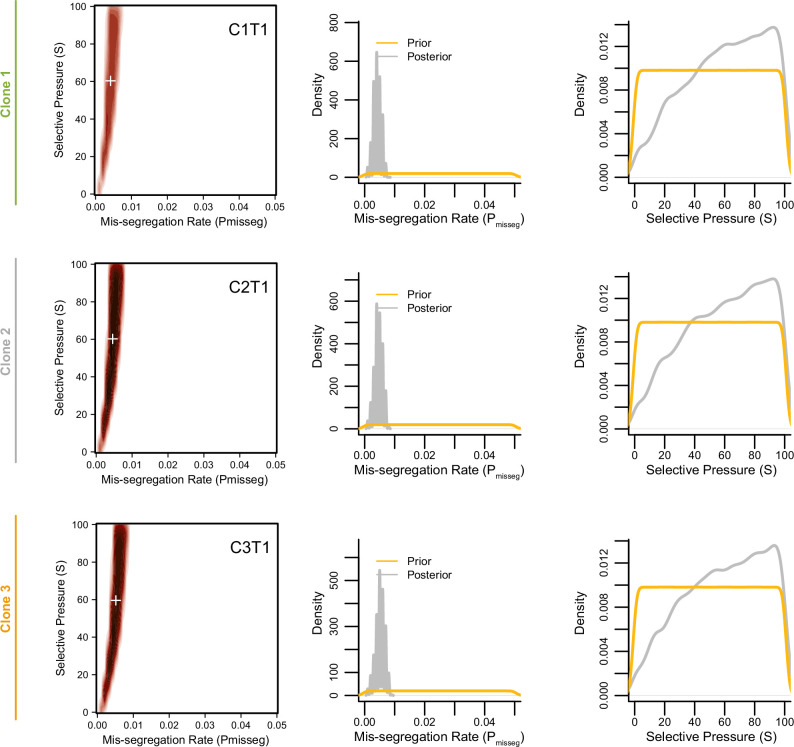
Joint posterior distributions from CRC organoids at 3 weeks. Joint (2D density plots) and individual (1D density plots) distributions of mis-segregation rates and selective pressures in individual clones of a patient-derived CRC organoid line from Bolhaqueiro et al., 2019 after 3 weeks of growth (accompanying data in Figure 6—figure supplement 4). The prior (yellow) distribution represents the parameters used for simulation while the posterior (gray) distribution represents the parameters from simulations whose observed measurements were similar to the measurements taken from the patient-derived sample using a tolerance threshold of 0.05. White + signs on joint distributions represent the mean of both parameters.

**Table 3.** Model selection.

Sample	Growt Model	Selectio Model	PP	BF (Ho Neutral)	Pmisseg	S	Steps
7T	exponential pseudo-Moran	Abundance	0.621	Inf	0.0033 ± 1e-05	60.5416 ± 0.2053	59.8475 ± 0.0937
7T	exponential pseudo-Moran	Driver	0.14	Inf	0.001 ± 1e-05	49.6557 ± 0.2389	58.7002 ± 0.0943
7T	exponential pseudo-Moran	Hybrid	0.239	Inf	8e-04 ± 1e-05	49.3428 ± 0.2377	58.5789 ± 0.0935
7T	exponential pseudo-Moran	Neutral	0	NA	9e-04 ± 5e-05	0 ± 0	57.7994 ± 0.6728
7T	constant Wright-Fisher	Abundance	0.985	Inf	0.0062 ± 2e-05	69.7026 ± 0.1724	59.9318 ± 0.0937
7T	constant Wright-Fisher	Driver	0	NA	0.0012 ± 1e-05	48.2881 ± 0.2384	57.5239 ± 0.0933
7T	constant Wright-Fisher	Hybrid	0.015	Inf	9e-04 ± 1e-05	50.7803 ± 0.2359	58.2514 ± 0.0941
7T	constant Wright-Fisher	Neutral	0	NA	9e-04 ± 5e-05	0 ± 0	58.7803 ± 0.6701
U1T	exponential pseudo-Moran	Abundance	0.582	199	9e-04 ± 1e-05	56.8672 ± 0.2168	59.9906 ± 0.0937
U1T	exponential pseudo-Moran	Driver	0.113	39	0.001 ± 1e-05	49.6611 ± 0.2389	58.6886 ± 0.0944
U1T	exponential pseudo-Moran	Hybrid	0.156	54	8e-04 ± 1e-05	49.3658 ± 0.2375	58.569 ± 0.0935
U1T	exponential pseudo-Moran	Neutral	0.149	1	9e-04 ± 5e-05	0 ± 0	57.7102 ± 0.67
U1T	constant Wright-Fisher	Abundance	0.654	290	0.001 ± 1e-05	61.4358 ± 0.2029	60.0021 ± 0.0937
U1T	constant Wright-Fisher	Driver	0.115	51	0.0012 ± 1e-05	48.2767 ± 0.2383	57.5267 ± 0.0934
U1T	constant Wright-Fisher	Hybrid	0.115	51	9e-04 ± 1e-05	50.8033 ± 0.2358	58.2507 ± 0.0941
U1T	constant Wright-Fisher	Neutral	0.115	1	9e-04 ± 5e-05	0 ± 0	58.7803 ± 0.6701
U2T	exponential pseudo-Moran	Abundance	0.628	251	0.0054 ± 1e-05	59.4269 ± 0.2108	59.8349 ± 0.0935
U2T	exponential pseudo-Moran	Driver	0.079	32	0.0027 ± 2e-05	50.1513 ± 0.2396	57.4538 ± 0.0934
U2T	exponential pseudo-Moran	Hybrid	0.166	66	0.0022 ± 2e-05	48.7779 ± 0.2413	57.7078 ± 0.0934
U2T	exponential pseudo-Moran	Neutral	0.127	1	0.0021 ± 7e-05	0 ± 0	56.8535 ± 0.6619
U2T	constant Wright-Fisher	Abundance	0.918	2817	0.0112 ± 3e-05	69.7222 ± 0.1703	60.0655 ± 0.0934
U2T	constant Wright-Fisher	Driver	0.001	4	0.0027 ± 2e-05	48.7794 ± 0.2389	56.4812 ± 0.0919
U2T	constant Wright-Fisher	Hybrid	0.064	196	0.0022 ± 1e-05	50.9564 ± 0.2379	57.1161 ± 0.0925
U2T	constant Wright-Fisher	Neutral	0.017	1	0.0022 ± 1e-04	0 ± 0	57.7898 ± 0.6841
U3T	exponential pseudo-Moran	Abundance	0.582	199	0.0029 ± 1e-05	60.9557 ± 0.2091	59.8273 ± 0.0938
U3T	exponential pseudo-Moran	Driver	0.113	39	0.001 ± 1e-05	49.6707 ± 0.2389	58.6986 ± 0.0944
U3T	exponential pseudo-Moran	Hybrid	0.156	54	8e-04 ± 1e-05	49.3754 ± 0.2376	58.5711 ± 0.0935
U3T	exponential pseudo-Moran	Neutral	0.149	1	9e-04 ± 5e-05	0 ± 0	57.7102 ± 0.67
U3T	constant Wright-Fisher	Abundance	0.736	Inf	0.0052 ± 2e-05	69.8357 ± 0.1713	59.932 ± 0.0934
U3T	constant Wright-Fisher	Driver	0.13	Inf	0.0012 ± 1e-05	48.2864 ± 0.2383	57.5385 ± 0.0934
U3T	constant Wright-Fisher	Hybrid	0.134	Inf	9e-04 ± 1e-05	50.8219 ± 0.2357	58.2482 ± 0.0941
U3T	constant Wright-Fisher	Neutral	0	NA	9e-04 ± 5e-05	0 ± 0	58.8567 ± 0.6676
14T	exponential pseudo-Moran	Abundance	0.582	199	9e-04 ± 1e-05	56.8672 ± 0.2168	59.9906 ± 0.0937
14T	exponential pseudo-Moran	Driver	0.113	39	0.001 ± 1e-05	49.6614 ± 0.239	58.695 ± 0.0944
14T	exponential pseudo-Moran	Hybrid	0.156	54	8e-04 ± 1e-05	49.3716 ± 0.2375	58.5632 ± 0.0935
14T	exponential pseudo-Moran	Neutral	0.149	1	9e-04 ± 5e-05	0 ± 0	57.7102 ± 0.67
14T	constant Wright-Fisher	Abundance	0.654	290	0.0011 ± 1e-05	62.8579 ± 0.2075	60.0029 ± 0.0936
14T	constant Wright-Fisher	Driver	0.115	51	0.0012 ± 1e-05	48.2967 ± 0.2383	57.5295 ± 0.0934
14T	constant Wright-Fisher	Hybrid	0.115	51	9e-04 ± 1e-05	50.8274 ± 0.2357	58.2478 ± 0.0941
14T	constant Wright-Fisher	Neutral	0.115	1	9e-04 ± 5e-05	0 ± 0	58.8567 ± 0.6676
16T	exponential pseudo-Moran	Abundance	0.582	199	0.002 ± 1e-05	61.2401 ± 0.2028	59.9109 ± 0.0935
16T	exponential pseudo-Moran	Driver	0.113	39	0.001 ± 1e-05	49.6539 ± 0.2389	58.7006 ± 0.0943
16T	exponential pseudo-Moran	Hybrid	0.156	54	8e-04 ± 1e-05	49.3611 ± 0.2376	58.574 ± 0.0935
16T	exponential pseudo-Moran	Neutral	0.149	1	9e-04 ± 5e-05	0 ± 0	57.7994 ± 0.6728
16T	constant Wright-Fisher	Abundance	0.654	290	0.0038 ± 1e-05	69.8456 ± 0.1701	59.9523 ± 0.0936
16T	constant Wright-Fisher	Driver	0.115	51	0.0012 ± 1e-05	48.261 ± 0.2384	57.5233 ± 0.0933
16T	constant Wright-Fisher	Hybrid	0.115	51	9e-04 ± 1e-05	50.7713 ± 0.2359	58.2554 ± 0.0941
16T	constant Wright-Fisher	Neutral	0.115	1	9e-04 ± 5e-05	0 ± 0	58.7803 ± 0.6701
19Ta	exponential pseudo-Moran	Abundance	0.711	313	0.004 ± 1e-05	60.6391 ± 0.2074	59.7801 ± 0.0934
19Ta	exponential pseudo-Moran	Driver	0.038	17	0.0028 ± 2e-05	50.2185 ± 0.2399	57.3764 ± 0.0934
19Ta	exponential pseudo-Moran	Hybrid	0.135	59	0.0022 ± 3e-05	48.3823 ± 0.242	57.5368 ± 0.0935
19Ta	exponential pseudo-Moran	Neutral	0.116	1	0.0022 ± 9e-05	0 ± 0	56.5955 ± 0.6549
19Ta	constant Wright-Fisher	Abundance	0.97	11760	0.0075 ± 2e-05	69.3863 ± 0.1735	59.956 ± 0.0938
19Ta	constant Wright-Fisher	Driver	0	0	0.0028 ± 2e-05	48.8413 ± 0.2392	56.4529 ± 0.0917
19Ta	constant Wright-Fisher	Hybrid	0.026	315	0.0023 ± 1e-05	50.8588 ± 0.2383	57.1031 ± 0.0925
19Ta	constant Wright-Fisher	Neutral	0.004	1	0.0023 ± 1e-04	0 ± 0	57.9522 ± 0.6869
19Tb	exponential pseudo-Moran	Abundance	0.727	320	0.0036 ± 1e-05	60.5885 ± 0.2085	59.829 ± 0.0938
19Tb	exponential pseudo-Moran	Driver	0.03	13	0.001 ± 1e-05	49.6622 ± 0.2389	58.6929 ± 0.0944
19Tb	exponential pseudo-Moran	Hybrid	0.127	56	8e-04 ± 1e-05	48.5237 ± 0.2322	58.9663 ± 0.0931
19Tb	exponential pseudo-Moran	Neutral	0.116	1	9e-04 ± 5e-05	0 ± 0	57.7102 ± 0.67
19Tb	constant Wright-Fisher	Abundance	0.979	47320	0.0068 ± 2e-05	69.5697 ± 0.173	59.9232 ± 0.0935
19Tb	constant Wright-Fisher	Driver	0	0	0.0012 ± 1e-05	48.2786 ± 0.2383	57.5433 ± 0.0934
19Tb	constant Wright-Fisher	Hybrid	0.02	982	9e-04 ± 1e-05	50.8162 ± 0.2357	58.2495 ± 0.0941
19Tb	constant Wright-Fisher	Neutral	0.001	1	9e-04 ± 5e-05	0 ± 0	58.8376 ± 0.669
24Ta	exponential pseudo-Moran	Abundance	0.731	321	0.0036 ± 1e-05	60.5303 ± 0.2082	59.8208 ± 0.0938
24Ta	exponential pseudo-Moran	Driver	0.029	13	0.001 ± 1e-05	49.6703 ± 0.2389	58.6938 ± 0.0944
24Ta	exponential pseudo-Moran	Hybrid	0.125	55	8e-04 ± 1e-05	49.3669 ± 0.2376	58.5778 ± 0.0935
24Ta	exponential pseudo-Moran	Neutral	0.116	1	9e-04 ± 5e-05	0 ± 0	57.7102 ± 0.67
24Ta	constant Wright-Fisher	Abundance	0.979	47346	0.0068 ± 2e-05	69.6173 ± 0.173	59.933 ± 0.0934
24Ta	constant Wright-Fisher	Driver	0	0	0.0012 ± 1e-05	48.2789 ± 0.2383	57.5377 ± 0.0934
24Ta	constant Wright-Fisher	Hybrid	0.02	956	9e-04 ± 1e-05	50.8229 ± 0.2357	58.2524 ± 0.0941
24Ta	constant Wright-Fisher	Neutral	0.001	1	9e-04 ± 5e-05	0 ± 0	58.8567 ± 0.6676
24Tb	exponential pseudo-Moran	Abundance	0.68	294	0.0046 ± 1e-05	60.2602 ± 0.2084	59.8073 ± 0.0936
24Tb	exponential pseudo-Moran	Driver	0.054	23	0.0031 ± 3e-05	50.2981 ± 0.2399	57.2927 ± 0.0934
24Tb	exponential pseudo-Moran	Hybrid	0.149	65	0.0025 ± 4e-05	48.3833 ± 0.244	57.4236 ± 0.0936
24Tb	exponential pseudo-Moran	Neutral	0.118	1	0.0025 ± 0.00013	0 ± 0	56.7229 ± 0.6579
24Tb	constant Wright-Fisher	Abundance	0.954	7730	0.0215 ± 0.00011	33.6703 ± 0.2962	59.9064 ± 0.0937
24Tb	constant Wright-Fisher	Driver	0	2	0.003 ± 2e-05	48.7528 ± 0.2393	56.4175 ± 0.0918
24Tb	constant Wright-Fisher	Hybrid	0.039	318	0.0024 ± 2e-05	50.7006 ± 0.2389	57.107 ± 0.0925
24Tb	constant Wright-Fisher	Neutral	0.006	1	0.0024 ± 0.00011	0 ± 0	58.0318 ± 0.6822
26N	exponential pseudo-Moran	Abundance	0.582	199	0.0021 ± 1e-05	60.9877 ± 0.2031	59.9205 ± 0.0934
26N	exponential pseudo-Moran	Driver	0.113	39	0.001 ± 1e-05	49.6389 ± 0.2389	58.7018 ± 0.0944
26N	exponential pseudo-Moran	Hybrid	0.156	54	8e-04 ± 1e-05	49.3389 ± 0.2377	58.5755 ± 0.0935
26N	exponential pseudo-Moran	Neutral	0.149	1	9e-04 ± 5e-05	0 ± 0	57.7994 ± 0.6728
26N	constant Wright-Fisher	Abundance	0.654	290	0.0039 ± 1e-05	69.794 ± 0.1704	59.9547 ± 0.0935
26N	constant Wright-Fisher	Driver	0.115	51	0.0012 ± 1e-05	48.2849 ± 0.2384	57.5175 ± 0.0933
26N	constant Wright-Fisher	Hybrid	0.115	51	9e-04 ± 1e-05	50.737 ± 0.2359	58.2609 ± 0.0941
26N	constant Wright-Fisher	Neutral	0.115	1	9e-04 ± 5e-05	0 ± 0	58.7803 ± 0.6701
9T	exponential pseudo-Moran	Abundance	0.685	299	0.0044 ± 1e-05	60.2829 ± 0.2086	59.7955 ± 0.0936
9T	exponential pseudo-Moran	Driver	0.052	23	0.0029 ± 2e-05	50.2323 ± 0.2398	57.3657 ± 0.0934
9T	exponential pseudo-Moran	Hybrid	0.147	64	0.0022 ± 3e-05	48.3829 ± 0.2422	57.5193 ± 0.0936
9T	exponential pseudo-Moran	Neutral	0.117	1	0.0023 ± 9e-05	0 ± 0	56.6083 ± 0.6581
9T	constant Wright-Fisher	Abundance	0.958	9299	0.0087 ± 2e-05	69.6836 ± 0.1724	59.926 ± 0.0937
9T	constant Wright-Fisher	Driver	0	1	0.0028 ± 2e-05	48.8394 ± 0.2392	56.4465 ± 0.0917
9T	constant Wright-Fisher	Hybrid	0.037	360	0.0023 ± 1e-05	50.8477 ± 0.2384	57.0952 ± 0.0925
9T	constant Wright-Fisher	Neutral	0.005	1	0.0023 ± 1e-04	0 ± 0	57.9427 ± 0.687
PolyB1	exponential pseudo-Moran	Abundance	0.635	261	0.0053 ± 1e-05	59.5088 ± 0.2104	59.8379 ± 0.0935
PolyB1	exponential pseudo-Moran	Driver	0.076	31	0.0028 ± 2e-05	50.2364 ± 0.2398	57.4025 ± 0.0934
PolyB1	exponential pseudo-Moran	Hybrid	0.164	67	0.0022 ± 3e-05	48.6949 ± 0.2419	57.6322 ± 0.0934
PolyB1	exponential pseudo-Moran	Neutral	0.124	1	0.0022 ± 9e-05	0 ± 0	56.5955 ± 0.6549
PolyB1	constant Wright-Fisher	Abundance	0.925	3482	0.0111 ± 3e-05	70.2557 ± 0.169	60.042 ± 0.0936
PolyB1	constant Wright-Fisher	Driver	0.001	4	0.0028 ± 2e-05	48.8194 ± 0.2391	56.4451 ± 0.0917
PolyB1	constant Wright-Fisher	Hybrid	0.061	228	0.0023 ± 1e-05	50.895 ± 0.2381	57.1073 ± 0.0925
PolyB1	constant Wright-Fisher	Neutral	0.014	1	0.0023 ± 1e-04	0 ± 0	57.9809 ± 0.6861
PolyB2	exponential pseudo-Moran	Abundance	0.603	218	0.0059 ± 1e-05	58.6612 ± 0.212	59.7835 ± 0.0937
PolyB2	exponential pseudo-Moran	Driver	0.086	31	0.0038 ± 4e-05	50.2948 ± 0.2394	57.0217 ± 0.093
PolyB2	exponential pseudo-Moran	Hybrid	0.17	61	0.004 ± 7e-05	48.9466 ± 0.2472	57.28 ± 0.0942
PolyB2	exponential pseudo-Moran	Neutral	0.141	1	0.0033 ± 0.00022	0 ± 0	56.5732 ± 0.6597
PolyB2	constant Wright-Fisher	Abundance	0.893	1277	0.0301 ± 1e-04	3.0543 ± 0.0165	59.9142 ± 0.0936
PolyB2	constant Wright-Fisher	Driver	0.003	4	0.0034 ± 3e-05	48.7328 ± 0.2396	56.3664 ± 0.0917
PolyB2	constant Wright-Fisher	Hybrid	0.069	98	0.0027 ± 2e-05	50.3534 ± 0.2405	57.1445 ± 0.0928
PolyB2	constant Wright-Fisher	Neutral	0.036	1	0.0026 ± 0.00014	0 ± 0	58.1592 ± 0.6741

Having confirmed the summary statistics from these samples were within the space of the simulation data with our chosen priors (Figure 6B), we performed ABC analysis on these datasets to infer rates of chromosome mis-segregation and levels of selection pressure and display the joint posterior distributions as 2D density plots (Figure 6C and D; Figure 6—figure supplements 2 and 3). Figure 6C illustrates the results for two individual colon organoid lines, showing the distribution of parameters used for simulations that gave the most similar results. With ABC, inferred parameters fall within rates of mis-segregation of about 0.001–0.006. Applied to a near-diploid cell, this translates to a range of about 5–38% of cell divisions having one chromosome mis-segregation. Importantly, these inferred rates of chromosome mis-segregation fall within the range of approximated *per chromosome* rates experimentally observed in cancer cell lines and human tumors (Figure 6E;Table 4, Table 5; Bakhoum et al., 2014; Bakhoum et al., 2011; Bakhoum et al., 2009; Dewhurst et al., 2014; Nicholson et al., 2015; Orr et al., 2016; Thompson and Compton, 2008; Worrall et al., 2018; Zasadil et al., 2014). Higher inferred mis-segregation rates tended to coincide with lower inferred selection experienced in these samples (Figure 6F). Posterior distributions in these samples were skewed toward high selection (S) indicating the presence stabilizing selection in all cases, where the average of the distributions of some samples were slightly lower or higher (Figure 6—figure supplement 3).

**Table 4.** Model selection with selective pressure constrained to S = 1.

Sample	Growth Model	Selection Model	PP	BF (Ho Neutral)	Pmisseg	S	Steps
7T	exponential pseudo-Moran	Abundance	0.274	1	9e-04 ± 5e-05	1 ± 0	58.2452 ± 0.6646
7T	exponential pseudo-Moran	Driver	0.238	1	9e-04 ± 5e-05	1 ± 0	58.4745 ± 0.6725
7T	exponential pseudo-Moran	Hybrid	0.26	1	9e-04 ± 5e-05	1 ± 0	58.586 ± 0.6668
7T	exponential pseudo-Moran	Neutral	0.228	1	9e-04 ± 6e-05	1 ± 0	58.5446 ± 0.6791
7T	constant Wright-Fisher	Abundance	0.259	1	9e-04 ± 6e-05	1 ± 0	58.8089 ± 0.6627
7T	constant Wright-Fisher	Driver	0.24	1	9e-04 ± 6e-05	1 ± 0	58.1783 ± 0.6771
7T	constant Wright-Fisher	Hybrid	0.257	1	9e-04 ± 5e-05	1 ± 0	59.0924 ± 0.6742
7T	constant Wright-Fisher	Neutral	0.245	1	9e-04 ± 7e-05	1 ± 0	58.7516 ± 0.6787
U1T	exponential pseudo-Moran	Abundance	0.275	1	9e-04 ± 5e-05	1 ± 0	58.2452 ± 0.6646
U1T	exponential pseudo-Moran	Driver	0.239	1	9e-04 ± 5e-05	1 ± 0	58.4745 ± 0.6725
U1T	exponential pseudo-Moran	Hybrid	0.258	1	9e-04 ± 5e-05	1 ± 0	58.586 ± 0.6668
U1T	exponential pseudo-Moran	Neutral	0.228	1	9e-04 ± 6e-05	1 ± 0	58.5446 ± 0.6791
U1T	constant Wright-Fisher	Abundance	0.259	1	9e-04 ± 6e-05	1 ± 0	58.8089 ± 0.6627
U1T	constant Wright-Fisher	Driver	0.24	1	9e-04 ± 6e-05	1 ± 0	58.1783 ± 0.6771
U1T	constant Wright-Fisher	Hybrid	0.257	1	9e-04 ± 5e-05	1 ± 0	59.1592 ± 0.6715
U1T	constant Wright-Fisher	Neutral	0.245	1	9e-04 ± 7e-05	1 ± 0	58.7516 ± 0.6787
U2T	exponential pseudo-Moran	Abundance	0.276	1	0.0021 ± 8e-05	1 ± 0	57.3057 ± 0.653
U2T	exponential pseudo-Moran	Driver	0.235	1	0.0024 ± 0.00011	1 ± 0	57.7452 ± 0.6634
U2T	exponential pseudo-Moran	Hybrid	0.264	1	0.0021 ± 7e-05	1 ± 0	58.1274 ± 0.654
U2T	exponential pseudo-Moran	Neutral	0.225	1	0.0024 ± 0.00011	1 ± 0	57.8758 ± 0.6772
U2T	constant Wright-Fisher	Abundance	0.269	1	0.0023 ± 1e-04	1 ± 0	58.3439 ± 0.6532
U2T	constant Wright-Fisher	Driver	0.233	1	0.0023 ± 9e-05	1 ± 0	57.4777 ± 0.693
U2T	constant Wright-Fisher	Hybrid	0.263	1	0.0023 ± 1e-04	1 ± 0	57.8662 ± 0.6683
U2T	constant Wright-Fisher	Neutral	0.236	1	0.0025 ± 0.00012	1 ± 0	57.1433 ± 0.6655
U3T	exponential pseudo-Moran	Abundance	0.275	1	9e-04 ± 5e-05	1 ± 0	58.1624 ± 0.6643
U3T	exponential pseudo-Moran	Driver	0.239	1	9e-04 ± 5e-05	1 ± 0	58.4554 ± 0.6736
U3T	exponential pseudo-Moran	Hybrid	0.258	1	9e-04 ± 5e-05	1 ± 0	58.586 ± 0.6668
U3T	exponential pseudo-Moran	Neutral	0.228	1	9e-04 ± 6e-05	1 ± 0	58.6178 ± 0.6777
U3T	constant Wright-Fisher	Abundance	0.259	1	9e-04 ± 6e-05	1 ± 0	58.7611 ± 0.6614
U3T	constant Wright-Fisher	Driver	0.24	1	9e-04 ± 6e-05	1 ± 0	58.1783 ± 0.6771
U3T	constant Wright-Fisher	Hybrid	0.257	1	9e-04 ± 5e-05	1 ± 0	59.0955 ± 0.674
U3T	constant Wright-Fisher	Neutral	0.245	1	9e-04 ± 7e-05	1 ± 0	58.7516 ± 0.6787
14T	exponential pseudo-Moran	Abundance	0.275	1	9e-04 ± 5e-05	1 ± 0	58.1624 ± 0.6643
14T	exponential pseudo-Moran	Driver	0.239	1	9e-04 ± 5e-05	1 ± 0	58.4554 ± 0.6736
14T	exponential pseudo-Moran	Hybrid	0.258	1	9e-04 ± 5e-05	1 ± 0	58.586 ± 0.6668
14T	exponential pseudo-Moran	Neutral	0.228	1	9e-04 ± 6e-05	1 ± 0	58.5446 ± 0.6791
14T	constant Wright-Fisher	Abundance	0.259	1	9e-04 ± 6e-05	1 ± 0	58.8089 ± 0.6627
14T	constant Wright-Fisher	Driver	0.24	1	9e-04 ± 6e-05	1 ± 0	58.1783 ± 0.6771
14T	constant Wright-Fisher	Hybrid	0.257	1	9e-04 ± 5e-05	1 ± 0	59.0924 ± 0.6739
14T	constant Wright-Fisher	Neutral	0.245	1	9e-04 ± 7e-05	1 ± 0	58.7516 ± 0.6787
16T	exponential pseudo-Moran	Abundance	0.274	1	9e-04 ± 5e-05	1 ± 0	58.2452 ± 0.6646
16T	exponential pseudo-Moran	Driver	0.238	1	9e-04 ± 5e-05	1 ± 0	58.4745 ± 0.6725
16T	exponential pseudo-Moran	Hybrid	0.26	1	9e-04 ± 5e-05	1 ± 0	58.586 ± 0.6668
16T	exponential pseudo-Moran	Neutral	0.228	1	0.001 ± 6e-05	1 ± 0	58.6274 ± 0.6789
16T	constant Wright-Fisher	Abundance	0.259	1	9e-04 ± 6e-05	1 ± 0	58.8089 ± 0.6627
16T	constant Wright-Fisher	Driver	0.24	1	9e-04 ± 6e-05	1 ± 0	58.1783 ± 0.6771
16T	constant Wright-Fisher	Hybrid	0.257	1	9e-04 ± 5e-05	1 ± 0	59.1051 ± 0.6742
16T	constant Wright-Fisher	Neutral	0.245	1	9e-04 ± 7e-05	1 ± 0	58.7516 ± 0.6787
19Ta	exponential pseudo-Moran	Abundance	0.273	1	0.0021 ± 8e-05	1 ± 0	57.4045 ± 0.6565
19Ta	exponential pseudo-Moran	Driver	0.243	1	0.0024 ± 0.00011	1 ± 0	57.8025 ± 0.663
19Ta	exponential pseudo-Moran	Hybrid	0.261	1	0.0022 ± 8e-05	1 ± 0	57.9108 ± 0.65
19Ta	exponential pseudo-Moran	Neutral	0.222	1	0.0025 ± 0.00012	1 ± 0	57.9331 ± 0.6777
19Ta	constant Wright-Fisher	Abundance	0.27	1	0.0024 ± 0.00011	1 ± 0	58.2866 ± 0.6566
19Ta	constant Wright-Fisher	Driver	0.233	1	0.0023 ± 1e-04	1 ± 0	57.8185 ± 0.6927
19Ta	constant Wright-Fisher	Hybrid	0.261	1	0.0023 ± 1e-04	1 ± 0	58.0478 ± 0.6705
19Ta	constant Wright-Fisher	Neutral	0.237	1	0.0025 ± 0.00012	1 ± 0	57.2261 ± 0.6669
19Tb	exponential pseudo-Moran	Abundance	0.275	1	9e-04 ± 5e-05	1 ± 0	58.1624 ± 0.6643
19Tb	exponential pseudo-Moran	Driver	0.239	1	9e-04 ± 5e-05	1 ± 0	58.4554 ± 0.6736
19Tb	exponential pseudo-Moran	Hybrid	0.258	1	9e-04 ± 5e-05	1 ± 0	58.586 ± 0.6668
19Tb	exponential pseudo-Moran	Neutral	0.228	1	9e-04 ± 6e-05	1 ± 0	58.5796 ± 0.6796
19Tb	constant Wright-Fisher	Abundance	0.259	1	9e-04 ± 6e-05	1 ± 0	58.7611 ± 0.6614
19Tb	constant Wright-Fisher	Driver	0.24	1	9e-04 ± 6e-05	1 ± 0	58.1178 ± 0.679
19Tb	constant Wright-Fisher	Hybrid	0.257	1	9e-04 ± 5e-05	1 ± 0	59.1592 ± 0.6715
19Tb	constant Wright-Fisher	Neutral	0.245	1	9e-04 ± 7e-05	1 ± 0	58.7516 ± 0.6787
24Ta	exponential pseudo-Moran	Abundance	0.275	1	9e-04 ± 5e-05	1 ± 0	58.1624 ± 0.6643
24Ta	exponential pseudo-Moran	Driver	0.239	1	9e-04 ± 5e-05	1 ± 0	58.4554 ± 0.6736
24Ta	exponential pseudo-Moran	Hybrid	0.258	1	9e-04 ± 5e-05	1 ± 0	58.586 ± 0.6668
24Ta	exponential pseudo-Moran	Neutral	0.228	1	9e-04 ± 6e-05	1 ± 0	58.6656 ± 0.6783
24Ta	constant Wright-Fisher	Abundance	0.259	1	9e-04 ± 6e-05	1 ± 0	58.7611 ± 0.6614
24Ta	constant Wright-Fisher	Driver	0.24	1	9e-04 ± 6e-05	1 ± 0	58.1783 ± 0.6771
24Ta	constant Wright-Fisher	Hybrid	0.257	1	9e-04 ± 5e-05	1 ± 0	59.1592 ± 0.6715
24Ta	constant Wright-Fisher	Neutral	0.245	1	9e-04 ± 7e-05	1 ± 0	58.7516 ± 0.6787
24Tb	exponential pseudo-Moran	Abundance	0.273	1	0.0023 ± 0.00011	1 ± 0	57.0446 ± 0.6526
24Tb	exponential pseudo-Moran	Driver	0.242	1	0.0025 ± 0.00012	1 ± 0	57.551 ± 0.6661
24Tb	exponential pseudo-Moran	Hybrid	0.264	1	0.0022 ± 9e-05	1 ± 0	57.9108 ± 0.6512
24Tb	exponential pseudo-Moran	Neutral	0.222	1	0.0026 ± 0.00013	1 ± 0	57.7516 ± 0.6758
24Tb	constant Wright-Fisher	Abundance	0.267	1	0.0024 ± 0.00013	1 ± 0	58.379 ± 0.6601
24Tb	constant Wright-Fisher	Driver	0.237	1	0.0024 ± 1e-04	1 ± 0	57.7357 ± 0.6922
24Tb	constant Wright-Fisher	Hybrid	0.257	1	0.0023 ± 1e-04	1 ± 0	57.9045 ± 0.6718
24Tb	constant Wright-Fisher	Neutral	0.239	1	0.0025 ± 0.00012	1 ± 0	57.2643 ± 0.6726
26N	exponential pseudo-Moran	Abundance	0.274	1	9e-04 ± 5e-05	1 ± 0	58.2452 ± 0.6646
26N	exponential pseudo-Moran	Driver	0.239	1	9e-04 ± 5e-05	1 ± 0	58.4045 ± 0.6706
26N	exponential pseudo-Moran	Hybrid	0.26	1	9e-04 ± 5e-05	1 ± 0	58.586 ± 0.6668
26N	exponential pseudo-Moran	Neutral	0.227	1	0.001 ± 7e-05	1 ± 0	58.6815 ± 0.6776
26N	constant Wright-Fisher	Abundance	0.259	1	9e-04 ± 6e-05	1 ± 0	58.8089 ± 0.6627
26N	constant Wright-Fisher	Driver	0.239	1	9e-04 ± 6e-05	1 ± 0	58.1783 ± 0.6771
26N	constant Wright-Fisher	Hybrid	0.257	1	9e-04 ± 5e-05	1 ± 0	59.1178 ± 0.6745
26N	constant Wright-Fisher	Neutral	0.245	1	0.001 ± 7e-05	1 ± 0	58.6879 ± 0.6762
9T	exponential pseudo-Moran	Abundance	0.274	1	0.0021 ± 8e-05	1 ± 0	57.3854 ± 0.6574
9T	exponential pseudo-Moran	Driver	0.242	1	0.0024 ± 0.00011	1 ± 0	57.8025 ± 0.663
9T	exponential pseudo-Moran	Hybrid	0.261	1	0.0022 ± 8e-05	1 ± 0	57.9108 ± 0.65
9T	exponential pseudo-Moran	Neutral	0.222	1	0.0025 ± 0.00012	1 ± 0	57.9522 ± 0.6787
9T	constant Wright-Fisher	Abundance	0.269	1	0.0024 ± 0.00011	1 ± 0	58.2866 ± 0.6566
9T	constant Wright-Fisher	Driver	0.233	1	0.0023 ± 1e-04	1 ± 0	57.9076 ± 0.6927
9T	constant Wright-Fisher	Hybrid	0.261	1	0.0023 ± 1e-04	1 ± 0	58.1115 ± 0.6708
9T	constant Wright-Fisher	Neutral	0.236	1	0.0025 ± 0.00012	1 ± 0	57.2261 ± 0.6669
PolyB1	exponential pseudo-Moran	Abundance	0.274	1	0.0021 ± 8e-05	1 ± 0	57.4045 ± 0.6565
PolyB1	exponential pseudo-Moran	Driver	0.243	1	0.0024 ± 0.00011	1 ± 0	57.7102 ± 0.6622
PolyB1	exponential pseudo-Moran	Hybrid	0.261	1	0.0022 ± 8e-05	1 ± 0	57.9459 ± 0.6512
PolyB1	exponential pseudo-Moran	Neutral	0.222	1	0.0025 ± 0.00011	1 ± 0	57.9522 ± 0.6776
PolyB1	constant Wright-Fisher	Abundance	0.271	1	0.0023 ± 0.00011	1 ± 0	58.2834 ± 0.6575
PolyB1	constant Wright-Fisher	Driver	0.231	1	0.0023 ± 9e-05	1 ± 0	57.6656 ± 0.6949
PolyB1	constant Wright-Fisher	Hybrid	0.261	1	0.0023 ± 1e-04	1 ± 0	57.9713 ± 0.6668
PolyB1	constant Wright-Fisher	Neutral	0.237	1	0.0025 ± 0.00012	1 ± 0	57.207 ± 0.6674
PolyB2	exponential pseudo-Moran	Abundance	0.272	1	0.0027 ± 2e-04	1 ± 0	56.8471 ± 0.6544
PolyB2	exponential pseudo-Moran	Driver	0.245	1	0.0029 ± 0.00021	1 ± 0	57.3312 ± 0.6609
PolyB2	exponential pseudo-Moran	Hybrid	0.263	1	0.0024 ± 0.00011	1 ± 0	57.9204 ± 0.6466
PolyB2	exponential pseudo-Moran	Neutral	0.221	1	0.0029 ± 0.00017	1 ± 0	57.4236 ± 0.6784
PolyB2	constant Wright-Fisher	Abundance	0.268	1	0.0025 ± 0.00013	1 ± 0	58.2484 ± 0.6616
PolyB2	constant Wright-Fisher	Driver	0.235	1	0.0026 ± 0.00014	1 ± 0	57.5796 ± 0.6897
PolyB2	constant Wright-Fisher	Hybrid	0.257	1	0.0026 ± 0.00015	1 ± 0	58.1115 ± 0.6741
PolyB2	constant Wright-Fisher	Neutral	0.24	1	0.0027 ± 0.00014	1 ± 0	57.379 ± 0.6701

**Table 5.** Approximate reported per chromosome mis-segregation rates.

1st Author	DOI	Model	Tumor?	Statistic	Assessment	Approximate observed frequency %	Aprrox modal chromosome # (ATCC)	Approximate mis-segregation rate (per chromosome)
Bakhoum	https://doi.org/10.1158/1078-0432.CCR-11-2049	Tumor-TMA	Tumor	Reported	Lagging/Bridging	31.3	46	0.00680
Orr	https://doi.org/10.1016/j.celrep.2016.10.030	U2OS	Tumor	Approx. Mean	Lagging	32.5	46	0.00707
Orr	https://doi.org/10.1016/j.celrep.2016.10.030	HeLa	Tumor	Approx. Mean	Lagging	22	82	0.00268
Orr	https://doi.org/10.1016/j.celrep.2016.10.030	SW-620	Tumor	Approx. Mean	Lagging	22.5	50	0.00450
Orr	https://doi.org/10.1016/j.celrep.2016.10.030	RPE1	Non-tumor	Approx. Mean	Lagging	2.5	46	0.00054
Orr	https://doi.org/10.1016/j.celrep.2016.10.030	BJ	Non-tumor	Approx. Mean	Lagging	8	46	0.00174
Nicholson	https://doi.org/10.7554/eLife.05068	Amniocyte	Non-tumor	Approx. Mean	Lagging	0	46	0.00000
Nicholson	https://doi.org/10.7554/eLife.05068	DLD1	Tumor	Approx. Mean	Lagging	1	46	0.00022
Dewhurst	https://doi.org/10.1158/2159-8290.CD-13-0285	HCT116-Diploid	Tumor	Approx. Mean	Lagging/Bridging	23	45	0.00511
Dewhurst	https://doi.org/10.1158/2159-8290.CD-13-0285	HCT116-Tetraploid	Tumor	Approx. Mean	Lagging/Bridging	50	90	0.00556
Bakhoum	https://doi.org/10.1038/ncb1809	U2OS	Tumor	Reported	Lagging		46	0.01000
Zasadil	https://doi.org/10.1126/scitranslmed.3007965	CAL51	Tumor	Approx. Mean	Lagging	0.5	44	0.00011
Thompson	https://doi.org/10.1083/jcb.200712029	RPE1	Non-tumor	Approx. Mean	Acute aneuploidy via FISH		46	0.00025
Thompson	https://doi.org/10.1083/jcb.200712029	HCT116-Diploid	Tumor	Approx. Mean	Acute aneuploidy via FISH		45	0.00025
Thompson	https://doi.org/10.1083/jcb.200712029	HT29	Tumor	Approx. Mean	Acute aneuploidy via FISH		71	0.00250
Thompson	https://doi.org/10.1083/jcb.200712029	Caco2	Tumor	Approx. Mean	Acute aneuploidy via FISH		96	0.00900
Thompson	https://doi.org/10.1083/jcb.200712029	MCF-7	Tumor	Approx. Mean	Acute aneuploidy via FISH		82	0.00700
Bakhoum	https://doi.org/10.1016/j.cub.2014.01.019	HCT116-Diploid	Tumor	Approx. Mean	Lagging	6	45	0.00133
Bakhoum	https://doi.org/10.1016/j.cub.2014.01.019	DLD1	Tumor	Approx. Mean	Lagging	2	46	0.00043
Bakhoum	https://doi.org/10.1016/j.cub.2014.01.019	HT29	Tumor	Approx. Mean	Lagging	14	71	0.00197
Bakhoum	https://doi.org/10.1016/j.cub.2014.01.019	SW-620	Tumor	Approx. Mean	Lagging	12	50	0.00240
Bakhoum	https://doi.org/10.1016/j.cub.2014.01.019	MCF-7	Tumor	Approx. Mean	Lagging	17	82	0.00207
Bakhoum	https://doi.org/10.1016/j.cub.2014.01.019	HeLa	Tumor	Approx. Mean	Lagging	13	82	0.00159
Worrall	https://doi.org/10.1016/j.celrep.2018.05.047	BJ	Non-tumor	Approx. Mean	Unspecified Error	5	46	0.00109
Worrall	https://doi.org/10.1016/j.celrep.2018.05.047	RPE1	Non-tumor	Approx. Mean	Unspecified Error	5	46	0.00109

To confirm the relevance of the inferred scalar exponent we performed our model selection scheme using only the simulation data with unmodified fitness values (S = 1; Table 4). In this case, we found that the inferred mis-segregation rates for most samples fell well below the expected range found in cancer cell lines (Figure 6E). Additionally, when we inferred mis-segregation rates and selection in the early timepoint of longitudinally sequenced organoid clones from Bolhaqueiro et al., 2019, the composition of the resultant populations simulated using these inferred characteristics better resembled the late-timepoint organoid data than those with unmodified selection values (S = 1; Figure 6—figure supplements 4 and 5).

As further validation for mis-segregation rates, we compared these inferred rates from CRC PDOs with those directly measured in live imaging from Bolhaqueiro et al., 2019. Although mis-segregation cannot be directly inferred from microscopy, diversity should correlate with the observed rate of mitotic errors. There was a strong correlation but for two outliers—14T and U1T (Figure 6G). In fact, when adjusting to the same scale and correcting for cell ploidy, these data follow a strong positive linear trend with a slightly lower slope than a 1:1 correlation, which could reflect an overestimation of mis-segregation rates in the microscopy data (Figure 6H). Particularly with lagging chromosomes, despite a chromosome’s involvement in an observed segregation defect, it may end up in the correct daughter cell. Overall, these results indicate that the inferred measures using approximate Bayesian computation and scDNAseq account for selection and provide a quantitative measure of CIN.

## Discussion

The clinical assessment of mutations, short indels, and microsatellite instability in human cancer determined by short-read sequencing currently guide clinical care. By contrast, CIN is highly prevalent, yet has remained largely intractable to clinical measures. Single-cell DNA sequencing now promises detailed karyotypic analysis across hundreds of cells, yet selective pressure suppresses the observed karyotype heterogeneity within a tumor. Optimal clinical measurement of CIN may be achieved with scDNAseq, but must additionally account for selective pressure, which reduces karyotype heterogeneity.

Despite the major limitations with current measures of CIN, emerging evidence hints at its utility as a biomarker to predict benefit to cancer therapy. For example, CIN measures appear to predict therapeutic response to paclitaxel (Janssen et al., 2009; Scribano et al., 2021; Swanton et al., 2009). Nevertheless, existing measures of CIN have had significant limitations. FISH and histological analysis of mitotic abnormalities are limited in quantifying specific chromosomes or requiring highly proliferative tumor types, such as lymphomas and leukemia. Gene expression profiles are proposed to correlate with CIN among populations of tumor samples (Carter et al., 2006), although they happen to correlate better with tumor proliferation (Sheltzer, 2013); in any case, they are correlations across populations of tumors, not suitable as an individualized diagnostic. We conclude that scDNAseq is the most complete and tractable measure of cellular karyotypes, and sampling at least 200 cells, coupled with computational models and ABC, promises to offer the best measure of tumor CIN.

Computational modeling of aneuploidy and CIN has been used to explore evolution in the context of numerical CIN and karyotype selection (Elizalde et al., 2018; Gao et al., 2016; Gusev et al., 2001; Gusev et al., 2000; Laughney et al., 2015; Nowak et al., 2002). Gusev and Nowak lay the foundation for mathematical modeling of CIN. While Gusev focused on the karyotypic outcomes of CIN, Nowak considered the effects of CIN-inducing mutations and the subsequent rate of LOH. Neither considered the individual fitness differences between specific karyotypes (Gusev et al., 2001; Gusev et al., 2000; Nowak et al., 2002). This was improved in Laughney et al., 2015 and Elizalde et al., 2018 where the authors leveraged the chromosome scores derived in Davoli et al., 2013, which enable the inclusion of oncogenes and tumor suppressors in models of CIN as we have done. These studies have provided important insights such as the role of whole-genome doubling as an evolutionary bridge to optimized chromosome stoichiometry. Yet the populations derived in these studies tend to vary to a greater degree than observed with scDNAseq, as they do not model strong selection against aneuploidy. Further, they do not attempt to use their models to measure CIN in biological samples. Here, we build on these models by considering, in addition to the selection on driver genes, the stabilizing selection wrought by chromosomal gene abundance. Further, we consider that the magnitude of selection pressure may not be a constant and implement a modifier to tune selection in our models. Lastly, we use our models as a quantitative measure of CIN that accounts for this selection.

Previous studies using single-cell sequencing identified surprisingly low karyotypic variance in human tumors including breast cancer (Gao et al., 2016; Kim et al., 2018; Wang et al., 2014) and colorectal and ovarian cancer organoids (Bolhaqueiro et al., 2019; Nelson et al., 2020). It has been difficult to understand these findings in the light of widespread CIN in human cancer (Sheltzer and Amon, 2011; Silk et al., 2013; Vasudevan et al., 2020; Weaver et al., 2007; Weaver and Cleveland, 2009). The best explanation of this apparent paradox is selection, which moderates karyotypic variance. Accounting for this, we can infer rates of chromosome mis-segregation in tumors or PDOs well within the range of rates observed microscopically in cancer cell lines. Additionally, no previous work, to our knowledge, has estimated the required sample size to infer CIN from scDNAseq data.

As described by others (Dewhurst et al., 2014; López et al., 2020), and consistent with our findings, early emergence of polyploid cells can markedly reduce apparent selection, leading to an elevated karyotype diversity over time. While we do not explicitly induce chance of whole genome doubling (WGD) events in simulations, populations that begin either diploid or tetraploid converge on near-triploid karyotypes over time, consistent with the notion that WGD can act as an evolutionary bridge to highly aneuploid karyotypes. Notably, our analysis indicates the samples with apparent polyploidy experienced among the lowest levels of karyotype selection.

In some early studies, CIN is considered a binary process—present or absent. We assumed that CIN measures are scalar, not binary, and measure this by rate of chromosome mis-segregation per division. A scalar is appropriate if, for example, there was a consistent probability of chromosome mis-segregation per division. However, we recognize that some mechanisms may not well adhere to this simplified model of CIN. For example, tumors with centrosome amplification may at times undergo bipolar division without mis-segregation, or, at other times, a multipolar division with extensive mis-segregation. Further, it is possible that some mechanisms may have correlated mis-segregations such that a daughter cell that gains one chromosome is more likely to gain other chromosomes, rather than lose them. Another possibility is that CIN could result in the mis-regulation of genes that further modify the rate of CIN. Our model does not yet account for punctuated behavior or changing rates of CIN. Furthermore, while recent studies have reported non-random mis-segregation of chromosomes (Dumont et al., 2020; Worrall et al., 2018), we did not incorporate these biases into our models as these studies do not reach consensus on which chromosomes are more frequently mis-segregated, which may be model-dependent.

Our approach reconstructs phylogenetic trees via copy number variation (CNV) analysis. This approach may be suboptimal given the selection on aneuploid states, and could be particularly problematic in the setting of convergent evolution. It is possible that this method results in low accuracy of the reconstructed phylogenies. Alternative approaches are possible, but would likely require re-design of the scDNAseq assay to include spiked-in primers that span highly polymorphic regions on each chromosome. If this were done, these sequences could be read in all cells and single-nucleotide polymorphisms could track individual maternal and paternal chromosomes, allowing a means of reconstructing cell phylogeny independent of CNVs. Despite this limitation, our phylogenetic reconstructions did seem to allow inference of CIN measures consistent with directly observed rates of chromosome mis-segregation in our taxol-induced CIN model as well as several independent cancer PDO models and cell lines.

A final limitation of our approach is we used previous estimates of cellular selection in our agent-based model and used these selection models to infer quantitative measures of CIN. While this approach seems to perform well in estimates of mis-segregation rates, we recognize that the selection models do not necessarily represent the real selective pressures on distinct aneuploidies. Future investigations are necessary to measure the selective pressure of distinct aneuploidies—a project that is now within technological reach. Selective pressures could also be influenced by cell type (Auslander et al., 2019; Dürrbaum et al., 2014; Sack et al., 2018; Starostik et al., 2020), tumor cell genetics (Foijer et al., 2014; Grim et al., 2012; López-García et al., 2017; Simões-Sousa et al., 2018; Soto et al., 2017), and the microenvironment (Hoevenaar et al., 2020).

In summation, we developed a theoretical and experimental framework for quantitative measure of chromosomal instability in human cancer. This framework accounts for selective pressure within tumors and employs Approximate Bayesian Computation, a commonly used analysis in evolutionary biology. Additionally, we determined that low-coverage single-cell DNA sequencing of at least 200 cells from a human tumor sample is sufficient to get an accurate ( > 90% accuracy) and reproducible measure of CIN. This work sets the stage for standardized quantitative measures of CIN that promise to clarify the underlying causes, consequences, and clinical utility of this nearly universal form of genomic instability.

## Materials and methods

### Agent-based modeling

Agent-based models were implemented using the agent-based platform, NetLogo 6.0.4 (Wilensky, 1999).

#### Underlying assumptions for models of CIN and karyotype selection

Chromosome mis-segregation rate is defined as the number of chromosome missegregation events that occur per cellular division.

Cell division always results in 2 daughter cells.

Pmisseg,c is assigned uniformly for each cell in a population and for each chromosome.

Cells die when the copy number of any chromosome is equal to 0 or exceeding 6 unless otherwise noted.

Steps are based on the rate of division of euploid cells. We assume a probability of division (Pdivision) of 0.5, or half of the population divides every step, for euploid populations. This probabilistic division is to mimic the asynchrony of cellular proliferation and to allow for positive selection, where some cells may divide more rapidly than their euploid ancestors.

No chromosome is more likely to mis-segregate than any other.

### Chromosome-arm scores

#### Gene abundance scores

The R package biomaRt v.2.46.3 was used to pull the chromosome arm location for each gene in Ensembl’s ‘Human genes’ dataset (GRCh38.p13). The number of genes on each chromosome arm were enumerated and Abundance scores were generated by normalizing the number of genes on each chromosome arm by the sum of all enumerated genes across chromosomes. Chromosome arms with no recorded genes were given a score of 0.

#### Driver density scores

Arm-level ‘TSG-OG-Ess’ scores derived in Davoli et al., 2013 were adapted for our purposes. These values were derived from a pan-cancer analysis (TCGA) of the frequency of mutation of these genes and their location in the genome. These scores correlate with the frequency with which chromosomes are found to be amplified in the genome. We adapted these scores by normalizing the published ‘TSG-OG-Ess’ score for each chromosome arm by the sum of all Charm scores. Chromosome arms with no published Charm score were given a score of 0. We refer to these as TOE scores for our purposes.

#### Hybrid scores

Chromosome arm scores for the Hybrid selection model are the average of the chromosome arm’s Gene Abundance and Driver Density scores.

### Implementing karyotype selection

In each model, numerical scores are assigned to each chromosome, the sum of which represents the fitness of the karyotype (Figure 1B). At each simulation time step, fitness is re-calculated for each cell based on its updated karyotype. These fitness values determine if they undergo mitosis in the next round. However, the modality of selection changes how those karyotypes are assessed. Here, we implement four separate karyotype selection models (1) gene abundance, (2) driver density, (3) a hybrid gene abundance and driver density, and (4) neutral selection. The scores that are generated in each produce a fitness value (F) that can then be subjected to pressure (S) as described above.

#### Selection on gene abundance

The Gene Abundance selection model relies on the concept of gene dosage stoichiometry where the aneuploid karyotypes are selected against and that the extent of negative selection scales with the severity of aneuploidy and the identity and gene abundance on the aneuploid chromosomes (Sheltzer and Amon, 2011). Chromosome arm fitness contribution scores (f_c_) are taken as the chromosome arm scores derived above (section 2.1) and the sum of these scores is 1. These base values are then modified under the gene abundance model to generate a contextual fitness score (CFS_GA,c_) at each time step such that…

$${\text{CFS}}_{\text{GA,c}}\text{=}{\text{f}}_{\text{c}}\text{-}\frac{{\text{f}}_{\text{c}}\times |{\text{n}}_{\text{c}}\text{-}{\overset{-}{\text{x}}}_{\text{p}}\text{|}}{{\overset{-}{\text{x}}}_{\text{p}}}$$

$$F=\sum_{c=1}^{46}{CFS}_{GA,c}$$

… where ${\bar{X}}_{p}$ is the average ploidy of the population and ${\text{n}}_{\text{c}}$ is the chromosome copy number. In this model, the fitness contribution of a chromosome declines as its distance from the average ploidy increases and that the magnitude of this effect is dependent on the size of the chromosome.

#### Selection on driver density

The Driver Density modality relies on assigned fitness values to chromosomes based on their relative density of tumor suppressor genes, essential genes, and oncogenes. Chromosome arm fitness contribution scores (f_c_) are taken as the chromosome arm scores derived above (section Driver density scores) and are employed such that…

$${\text{CFS}}_{\text{TOE,c}}\text{=}\frac{{\text{n}}_{\text{c}}\times {\text{TOE}}_{\text{c}}}{{\overset{-}{\text{x}}}_{\text{p}}}$$

$$\text{F=}\sum_{\text{c=1}}^{\text{46}}{\text{CFS}}_{\text{TOE,c}}$$

This selection model benefits cells that have maximized the density of oncogenes and essential genes to tumor suppressors through chromosome mis-segregation.

#### Hybrid selection

The hybrid model relies on selection on both gene abundance and driver densities. CFS_TOE,c_ and CFS_GA,c_ are both calculated and averaged such that…

$$\text{F = }\sum_{\text{c=1}}^{\text{46}}\frac{{\text{CFS}}_{\text{GA,c}}+{\text{CFS}}_{\text{TOE,c}}}{\text{2}}$$

#### Neutral selection

When populations are grown under neutral selection, the fitness of each cell is constitutively set to 1 regardless of the cells’ individual karyotypes.

F = 1

#### Scaling selection pressure

Within each model of karyotype selection, the magnitude of selective pressure upon any karyotype, with fitness F, can be scaled by applying the scalar exponent S to produce a modified fitness score F_M_. Thus…

$${\text{F}}_{\text{M}}\text{ = }{\text{F}}^{\text{S}}$$

For example, in the Gene Abundance model of karyotype selection, an otherwise diploid cell with three copies of chromosome 1 in a diploid population will have a F value of 0.954. Under selection-null conditions (S = 0)…

$${\text{F}}_{\text{M}}\text{=}{\text{F}}^{\text{S}}\text{=}{\text{0.954}}^{\text{0}}\text{=}1$$

… the fitness of the aneuploid cell is equivalent to that of a euploid cell. Under conditions of high selection (S = 50)…

$${\text{F}}_{\text{M}}\text{=}{\text{F}}^{\text{S}}\text{=}{\text{0.954}}^{\text{50}}\text{=}0.097$$

…fitness of the aneuploid cell is ~10% that of the euploid cell and thus divides ~10% as frequently.

### Modeling growing and constant population dynamics

To accommodate different population size dynamics, we implemented our model using either growing, pseudo-Moran limited population dynamics and constant-size populations with approximated Wright-Fisher population dynamics.

#### Simulating CIN in exponentially growing populations with pseudo-Moran limits

Populations begin with 100 founder cells with a euploid karyotype of integer value ${\bar{X}}_{p}$ and the simulation is initiated.

CFS values are calculated for each chromosome in a cell according to the chosen karyotype selection model.

Cellular fitness is calculated based on CFS values.

Selective pressure (S) is applied to fitness (F) values to modify cellular fitness (F_M_).

Cells are checked to see if any death conditions are met and if the population limit is met. Cells die if any chromosome arm copy (n_c_) is less than 1 or greater than 6 (unless otherwise indicated). We implemented population size limits in a pseudo-Moran fashion to reduce computational constraints. If the population size is 3000 cells or greater, a random half of the population is deleted.

Cells probabilistically divide if their fitness is greater than a random float (R) between 0 and 2. Thus...

R∼U[0,1]

If a cell does not divide, it restarts the cycle from CFS values are calculated for each chromosome in a cell according to the chosen karyotype selection model. If a cell divides, mis-segregations may occur.

Each copy (n_c_) of each chromosome (c) has an opportunity to mis-segregate probabilistically. For each chromosome copy, a mis-segregation occurs if a random float (R) between 0–1 falls below P_misseg_. Thus...

R∼U[0,1]

$$Mis-segregate\;chromosome\;c\;if\;{\text{P}}_{misseg,c>R}$$

If a chromosome copy is not mis-segregated, the next chromosome copy is tested. If a chromosome copy is mis-segregated, chromosome arms may be segregated separately (i.e. a reciprocal, arm-level CNA) if a random float (R) between 0 and 1 falls below P_break_. Thus...

R∼U[0,1]

$$Break\;chromosome\;c\;if\;P_{misseg,c>R}$$

The karyotype of the cell is modified according to the results of the mis-segregation sequence above. When the mis-segregation sequence is complete, a clone of the initial cell with any reciprocal copy number alterations to its karyotype is created.

The simulation ends if it reaches 100 steps and data are exported. Otherwise, the simulation continues from CFS values are calculated for each chromosome in a cell according to the chosen karyotype selection model.

#### Simulating CIN in constant-size populations with approximated Wright-Fisher dynamics

We approximated constant-size Wright-Fisher dynamics in our model by re-initiating the population at each time step and randomly drawing from the previous generation’s distribution of chromosome copy numbers for each chromosome in each cell of the new population. Because the exponential pseudo-Moran model relies on proliferation rates across over-lapping generations to enact karyotype selection, such a method would not be useful here. To accommodate karyotype selection in this model, we employed an additional baseline death rate of about 20% (Sottoriva et al., 2015) that increases for cells with lower fitness and decreases for cells with higher fitness (see section 4.2.9). In this way, the karyotypes of the cells that die are removed from the pool of karyotypes that are drawn upon in the subsequent generation. CIN is simulated in this model as follows:

Populations begin with 4,500 founder cells and the simulation is (re-)initiated. The population begins with a euploid karyotype of integer value ${\bar{X}}_{p}$ if the population is being created for the first time.

Cells divide every step, regardless of fitness.

Chromosomes are mis-segregated in the same fashion as the exponential pseudo-Moran model above (sections 4.1.8–4.1.10).

The simulation ends if it reaches 100 steps and data are exported. Otherwise, the simulation continues from 4.2.1.

CFS values are calculated for each chromosome in a cell according to the chosen karyotype selection model.

Cellular fitness is calculated based on CFS values.

Selective pressure (S) is applied to fitness (F) values to modify cellular fitness (F_M_).

Cells are checked to see if any death conditions are met and if the population limit is met. Cells die if any chromosome arm copy (n_c_) is less than 1 or greater than 6 (unless otherwise indicated).

Additionally, the cells’ fitness values and a random float (R) between 0 and 5 are used to determine if they die. In this way, a cell with a fitness of 1 has a 20% baseline death rate. Thus, cells die if…

$$\frac{\text{1}}{F^{S}+0.001}>R∼U[0,5]$$

After determining cell death, the copy number distributions of each cells’ chromosome arm (c) are individually stored.

The cycle repeats from 4.2.1. However, the re-initated population will have its chromosome arm copy numbers drawn from the previous generation’s stored chromosome arm copy number distributions.

### Analysis of population diversity and topology in biological and simulated data

Phylogenetic trees were reconstructed from chromosome copy number profiles from live and simulated cells by calculating pairwise Euclidean distance matrices and performing complete-linkage clustering in R (R Development Core Team, 2021). Phylogenetic tree topology measurements were performed in R using the package phyloTop v2.1.1 (Kendall et al., 2018). Sackin and Colless indices of tree imbalance were calculated, normalizing to the number of tree tips. Cherry and pitchfork number were also normalized to the size of the tree. MKV is taken as the variance of individual chromosomes taken across the population, averaged across all chromosomes, then normalized to the average ploidy of the population. Average aneuploidy is calculated as the variance within a single cell’s karyotype averaged across the population.

### Approximate bayesian computation

Approximate Bayesian computation was used for parameter inference of experimental data from simulated data. For this we employed the the “abc” function in the R package abc v2.1 (Csilléry et al., 2010). In short, a set of simulation parameters, *θ_i_*, is sampled from the prior distribution. This set of parameters corresponds to a set of simulated summary statistics, *S(y_i_*), in this case phylogenetic tree shapes, which can be compared to the set of experimental summary statistics, *S(y_o_*). The Euclidean distance between the experimental and simulated summary statistics can then be calculated (*dS(y_i_),S(y_o_*)). A threshold, *T,* is then selected—0.05 in our case—which rejects the lower 1 *T* sets of simulation parameters that correspond. The remaining parameters represent those that gave summary statistics with the highest similarity to the experimental summary statistics. These represent the posterior distribution of accepted parameters.

Bayesian model selection was performed using the “postpr” function in the same R package using tolerance threshold of 0.05 and rejection sampling method. This was used to calculate the posterior probability of each selection model *within* each growth model and the Bayes factor for each selection model with neutral selection as the null hypothesis. Bayes factors > 5 were considered substantial evidence of the alternative hypothesis.

### Sliding window analysis to tune time-steps for approximate Bayesian computation

We chose which simulation time steps to use for approximate Bayesian computation on organoid and biopsy data by repeating the inference using a sliding window of prior datasets with a width of 11 time steps (i.e. parameters from steps ∈ [0–10], [10-20], …, [91-100]) to see if the posterior distributions would stabilize over time. We then chose simulations from 40 to 80 time steps as our prior dataset as this range provided both a stable inference and is centered around 60 time steps (analogous to 30 generations, estimated to generate a 1 cm palpable mass of ~1 billion cells).

### Cell cultivation procedures

Cal51 cells expressing stably integrated RFP-tagged histone H2B and GFP-tagged a-tubulin were generated as previously described (Zasadil et al., 2014). Cells were maintained at 37 ºC and 5% CO_2_ in a humidified, water-jacketed incubator and propagated in Dulbecco’s Modified Eagle’s Medium (DMEM) – High Glucose formulation (Cat #: 11965118) supplemented with 10% fetal bovine serum and 100 units/mL penicillin-streptomycin. Paclitaxel (Tocris Bioscience, Cat #: 1097/10) used for cell culture experiments was dissolved in DMSO. The Cal51 cells were obtained from the DSMZ-German Collection of Microorganisms and Cell Cultures and were free from mycoplasma contamination prior to study. Karyotype analysis confirms the near-diploid characteristic of the cell line and the presence of both fluorescent markers suggests they are free of other contaminating cell lines.

### Time-lapse fluorescence microscopy

Cal51 cells were transduced with lentivirus expressing mNeonGreen-tubulin-P2A-H2B-FusionRed. A monoclonal line was treated with 20 nM paclitaxel for 24, 48, or 72 hr before timelapse analysis at 37 ^o^C and 10% CO_2_. Five 2 µm z-plane images were acquired using a Nikon Ti-E inverted microscope with a cMos camera at 3-min intervals using a 40 X/0.75 NA objective lens and Nikon Elements software.

### Flow cytometric analysis and cell sorting

Cells were harvested with trypsin, passed through a 35 μm mesh filter, and rinsed with PBS prior to fixation in ice cold 80% methanol. Fixed cells were stored at –80 ºC until analysis and sorting at which point fixed cells were resuspended in PBS containing 10 μg/ml DAPI for cell cycle analysis.

#### Flow cytometric analysis

Initial DNA content and cell cycle analyses were performed on a 5 laser BD LSR II. Doublets were excluded from analysis via standard FSC/SSC gating procedures. DNA content was analyzed via DAPI excitation at 355 nm and 450/50 emission using a 410 nm long pass dichroic filter.

#### Fluorescence activated cell sorting

Cell sorting was performed using the same analysis procedures described above on a BD FACS AriaII cell sorter. In general, single cells were sorted through a 130 μm low-pressure deposition nozzle into each well of a 96-well PCR plate containing 10 μl Lysis and Fragmentation Buffer cooled to 4 ºC on a Eppendorf PCR plate cooler. Immediately after sorting PCR plates were centrifuged at 300 x g for 60 s. For comparison of single-cell sequencing to bulk sequencing, 1000 cells were sorted into each ‘bulk’ well. The index of sorted cells was retained allowing for the post hoc estimation of DNA content for each cell.

### Low-coverage single-cell whole genome sequencing

Initial library preparation for low-coverage scDNAseq was performed as previously described (Leung et al., 2016) and adapted for low coverage whole genome sequencing instead of high coverage targeted sequencing. Initial genome amplification was performed using the GenomePlex Single Cell Whole Genome Amplification Kit and protocol (Sigma Aldrich, Cat #: WGA4). Cells were sorted into 10 μl pre-prepared Lysis and Fragmentation buffer containing Proteinase K. DNA was fragmented to an average of 1 kb in length prior to amplification. Single cell libraries were purified on a 96-well column plate (Promega, Cat #: A2271). Library fragment distribution was assessed via agarose gel electrophoresis and concentrations were measured on a Nanodrop 2000. Sequencing libraries were prepared using the QuantaBio sparQ DNA Frag and Library Prep Kit. Amplified single-cell DNA was enzymatically fragmented to ~250 bp, 5’-phosphorylated, and 3’-dA-tailed. Custom Illumina adapters with 96 unique 8 bp P7 index barcodes were ligated to individual libraries to enable multiplexed sequencing (Leung et al., 2016). Barcoded libraries were amplified following size selection via AxygenAxyPrep Mag beads (Cat #: 14-223-152). Amplified library DNA concentration was quantified using the Quant-iT Broad-Range dsDNA Assay Kit (Thermo, Cat #: Q33130). Single-cell libraries were pooled to 15 nM and final concentration was measured via qPCR. Single-end 100 bp sequencing was performed on an Illumina HiSeq2500.

### Single-cell copy number sequencing data processing

Single-cell DNA sequence reads were demultiplexed using unique barcode index sequences and trimmed to remove adapter sequences. Reads were aligned to GRCh38 using Bowtie2. Aligned BAM files were then processed using Ginkgo to make binned copy number calls. Reads are aligned within 500 kb bins and estimated DNA content for each cell, obtained by flow cytometric analysis, was used to calculate bin copy numbers based on the relative ratio of reads per bin (Garvin et al., 2015). We modified and ran Ginkgo locally to allow for the analysis of highly variable karyotypes with low ploidy values (see Code and Data Availability). Whole-chromosome copy number calls were calculated as the modal binned copy number across an individual chromosome. Cells with fewer than 100,000 reads were filtered out to ensure accurate copy number calls (Baslan et al., 2015). Cells whose predicted ploidy deviated more than 32% from the observed ploidy by FACS were also filtered out. The final coverage for the filtered dataset was 0.03 (5). Single cell data extracted from Navin et al., 2011 were separated into their individual clones and depleted of euploid cells. Single cell data from Bolhaqueiro et al., 2019 were filtered to include only the aneuploid data that fell within the ploidies observed in the study (see Code and Data Availability).

### Review and approximation of mis-segregation rates from published Studies

We reviewed the literature to extract per chromosome rates of mis-segregation for cell lines and clinical samples. Some studies publish these rates. For those that did not, we estimated these rates by approximating the plotted incidence of segregation errors thusly:

$$Approximate\;missegregration\;rate\;per\;chromosome=\frac{Observed\;\%\;frequency\;of\;errors\;per\;division/100}{Total\;\#\;modal\;chromosomes\;in\;sample}$$

Modal chromosome numbers were either taken from ATCC where available or were assumed to equal 46. Observed % frequencies were approximated from published plots. Approximated rates assume that 1 chromosome is mis-segregated at a time.

## Data Availability

Single-cell DNA sequencing data from this study has been deposited in NCBI SRA (PRJNA725515). All data and scripts used for modeling and analysis have been deposited in OSF at https://osf.io/snrg3/. The following datasets were generated: LynchAR ArpNL ZhouAS WeaverBA BurkardME 2021Quantifying chromosomal instability from intratumoral karyotype diversity using agent- based modeling and Bayesian inferenceOpen Science Framework10.17605/OSF.IO/SNRG3PMC905413235380536 LynchAR ArpNL ZhouAS WeaverBA BurkardME 2021Quantifying chromosomal instability from intratumoral karyotype diversity Quantifying chromosomal instability from intratumoral karyotype diversityNCBI BioProjectPRJNA72551510.7554/eLife.69799PMC905413235380536

## Additional files

Transparent reporting form
